# Clinical and genetic landscape of epilepsies with absence seizures and single‐gene etiology

**DOI:** 10.1111/epi.18655

**Published:** 2025-10-25

**Authors:** Simona Balestrini, Ilaria Galli, Maria Luisa Ricci, Elena Parrini, Davide Mei, Mario Mastrangelo, Francesco Pisani, Corinna Filippi, Lucio Giordano, Elisabetta Cesaroni, Carla Marini, Emanuele Cerulli Irelli, Carlo di Bonaventura, Marica Rubino, Antonietta Coppola, Jacopo Proietti, Tommaso Lo Barco, Francesca Darra, Laura Licchetta, Francesca Bisulli, Marco Perulli, Domenica Battaglia, Angela De Dominicis, Marina Trivisano, Nicola Specchio, Roberta Solazzi, Davide Caputo, Laura Canafoglia, LICE Collaborative group, Carmen Barba, Emanuele Bartolini, Pia Bernardo, Maria Paola Canevini, Gaetano Cantalupo, Susanna Casellato, Mara Cavallin, Ilaria Contaldo, Alessandro Ferretti, Maria Luigia Gambardella, Tiziana Granata, Giuliana Lentini, Anna Luchetti, Federico Melani, Pasquale Parisi, Simona Pellacani, Laura Pietrangelo, Tiziana Pisano, Ilaria Onida, Emilia Ricci, Aglaia Vignoli, Renzo Guerrini

**Affiliations:** ^1^ Neuroscience and Human Genetics Department Meyer Children’s Hospital IRCCS Florence Italy; ^2^ Department of Neuroscience, Pharmacology and Child Health University of Florence Florence Italy; ^3^ Department of Women/Child Health and Urological Science Sapienza University of Rome Rome Italy; ^4^ Unit of Child Neurology and Psychiatry, Department of Neuroscience/Mental Health Azienda Ospedaliero Universitaria Policlinico Umberto I‐ Rome Rome Italy; ^5^ Department of Human Neuroscience Sapienza University of Rome Rome Italy; ^6^ Paediatric Neurology and Psychiatry Unit, Spedali Civili Children’s Hospital University of Brescia Brescia Italy; ^7^ Child Neurology and Psychiatric Unit G. Salesi Pediatric Hospital; AOU Delle Marche Ancona Italy; ^8^ Department of Human Neurosciences Sapienza University Rome Italy; ^9^ Neurology Unit University Hospital Federico II Naples Italy; ^10^ Child Neuropsychiatric Unit, Department of Engineering for Innovation Medicine University of Verona Verona Italy; ^11^ IRCCS Istituto Delle Scienze Neurologiche di Bologna Bologna Italy; ^12^ Department of Biomedical and Neuromotor Sciences University of Bologna Bologna Italy; ^13^ Pediatric Neurology and Pschiatry, Dipartimento Della Salute Della Donna, Del Bambino e di Sanità Pubblica Fondazione Policlinico Universitario A. Gemelli, IRCCS Rome Italy; ^14^ Dipartimento di Scienza Della Vita e di Sanità Pubblica Università Cattolica del Sacro Cuore Rome Italy; ^15^ Neurology Epilepsy and Movement Disorders Unit Bambino Gesu’ Children’s Hospital, IRCCS Rome Italy; ^16^ University Hospitals KU Leuven Leuven Belgium; ^17^ Department of Pediatric Neuroscience Fondazione IRCCS Istituto Neurologico Carlo Besta Milan Italy

**Keywords:** atypical absence, epilepsy classification, monogenic, neurodevelopment, next‐generation sequencing

## Abstract

**Objective:**

To characterize the clinical, electroencephalographic, and genetic features of epilepsies featuring absence seizures within monogenic etiology, highlighting the diagnostic, treatment and prognostic implications.

**Methods:**

We conducted a retrospective, multicenter study including patients with monogenic epilepsies and electroencephalography (EEG)–documented absence seizures. We analyzed clinical data, electroclinical findings, neurodevelopmental outcomes, and treatment responses through standardized questionnaires and medical records. We classified genetic variants according to American College of Medical Genetics and Genomics (ACMG) guidelines and performed univariate and multivariate analyses to identify predictors of developmental outcomes.

**Results:**

We included 160 patients (111 female; median age at last follow‐up: 13 years) with absence seizures and confirmed pathogenic or likely pathogenic monogenic variants. The most frequently implicated genes were *SLC2A1, SLC6A1, SYNGAP1*, *CHD2,* and *SCN1A*. Four genes—*HESX1, NCKAP1, SON, STARD9*—had not been previously associated with absence seizures. In 35% of patients, absence seizures were the only seizure type and in 67% were the initial manifestation. Atypical features included irregular EEG discharges (56%) eyelid myoclonia (42%), and automatisms (33%). Early‐onset (before age 3) seizures occurred in 58% and was significantly associated with atypical features (*p* < .03). Using existing International League Against Epilepsy (ILAE) epilepsy syndrome classification, 60% of patients could not be classified. Developmental delay occurred in 54%, intellectual disability in 65%, and other neurodevelopmental comorbidities in 49%. Predictors of poor developmental outcomes included early developmental delay, drug‐resistant epilepsy, and early absence onset. We found no difference in the prevalence of drug resistance across the various genetic etiologies. The most effective medications for absence seizures included valproate, ethosuximide, benzodiazepines, and lamotrigine. Disease‐specific therapies (e.g., ketogenic diet in *SLC2A1*, stiripentol/fenfluramine in *SCN1A)* were effective in select cases.

**Significance:**

Absence seizures are a common manifestation of different monogenic epilepsies, often associated with early onset, atypical clinical and/or EEG features, developmental delay or drug resistance. Classification models should incorporate genetic data alongside electroclinical features, especially as next‐generation sequencing is increasingly used.

**Key points**

- Absence seizures in monogenic epilepsies often show early onset, atypical features, drug‐resistance, and poor developmental outcomes.
- *SLC2A1, SLC6A1, SYNGAP1, CHD2*, and *SCN1A* were the most frequent genes identified.
- Sixty percent of patients could not be classified within existing International League Against Epilepsy (ILAE) epilepsy syndromes.
- Genetic testing should be performed when absence seizures show atypical features, early onset, developmental delay, or drug resistance.

## 1 INTRODUCTION

Absence seizures occur in multiple idiopathic generalized epilepsy (IGE) syndromes, including childhood absence epilepsy (CAE), juvenile absence epilepsy (JAE), and juvenile myoclonic epilepsy (JME).^1^ IGEs comprise about 15%–20% of all epilepsies.^2^ Among IGEs, the frequency of CAE ranges from 1.5% to 12.1%, JAE from 0.2% to 2.4%, and JME from 5% to 10%.^2^ Atypical absence seizures occur in Lennox–Gastaut syndrome and can be present in other severe epilepsies including developmental and epileptic encephalopathies (DEEs),^3^ the most severe group of epilepsies, with onset typically in infancy or childhood. This category includes different epilepsy syndromes and patients who cannot be assigned to any specific syndrome but share a severe presentation and frequent comorbidities.^4^

Absence seizures, typical or atypical, are also observed in the so‐called genetic generalized epilepsies (GGEs), a broad group of generalized epilepsies, with definite or presumed genetic etiology. Because a severe form of GGE may also constitute a DEE and within the GGEs also include the IGE syndromes, among which CAE, JAE, JME, and epilepsy with generalized tonic–clonic seizures alone are the most common and usually have a good prognosis for seizure control, the term GGE is now considered as a broad category with limited specificity.^1^

Classically, the ictal semiology of typical absence seizures is a brief arrest of activity, accompanied by some combination of staring, blinking, or other eyelid movements, and automatisms of the mouth or limbs.^5^ The latest International League Against Epilepsy (ILAE) classification has removed the term “nonmotor” to define absence seizures,^6^ as motor features are at times observed during them.^1^ Absence seizures are clinically recognizable but there is variability of individual features between patients^7^ and the relationship between the variability in seizure semiology, other clinical features before treatment, and outcomes is unknown.

Since next‐generation sequencing (NGS) has become available, there has been an exponential growth of genes for “genes of major effect” that have been identified to be causative of monogenic epilepsies. More than 1000 monogenic etiologies have been identified, with a detection rate of potentially pathogenic variants in up to 50% of people with different types of epilepsy.^8^, ^9^ Almost 90% of monogenic etiologies are associated with DEEs. By comparison only 5% of “epilepsy genes” are associated with monogenic causes of “common epilepsies” (i.e., generalized and focal epilepsy syndromes).^9^

Although genetic testing is not recommended in most IGEs, a positive family history, additional symptoms (e.g. intellectual disability, dysmorphology, etc.), which may be mild and escape recognition, and atypical features (e.g., early onset, drug resistance) may support the search for a single‐gene etiology.^10^, ^11^, ^12^

In this study, we characterized the clinical and EEG phenotype in a large cohort of patients featuring absence seizures within a monogenic epilepsy syndrome, with the aim of better defining the phenotypic spectrum of monogenic epilepsies with absence seizures within the wider GGE category and strengthening the recommendations for NGS diagnostics in patients with absence seizures. We hypothesized that atypical features associated with absence seizures may serve as clinical markers for underlying monogenic etiologies.

## 2 PATIENTS AND METHODS

This retrospective, observational study was conducted across 17 Italian epilepsy centers with the support of the network of the Genetics Commission of the Italian League Against Epilepsy (LICE). We included patients according to the following criteria: typical or atypical absence seizures recorded on ictal electroencephalography (EEG), with electrographic confirmation of the seizure pattern, established monogenic etiology (i.e., likely pathogenic or pathogenic variant based on the American College of Medical Genetics and Genomics [ACMG] classification^13^). We excluded patients with variants of uncertain significance (VUS) or with a genetic etiology that was not monogenic (i.e., pathogenic copy number variant [CNV] or chromosomal abnormality). Data collection and analysis were approved by the local ethics committees of each participating institution; data were anonymized for analysis and merged into a single data set.

We defined patients' phenotypes through a neurological and epilepsy history as well as review of medical records. Using a standardized template, we conducted an evaluation of all seizure types (i.e., frequency, age at onset, response to treatment), EEG (i.e., background activity, epileptiform abnormalities while awake and asleep, photosensitivity, ictal findings), neuroimaging, and cognitive and behavioral comorbidities. Formal neuropsychological assessment was unavailable or not performed in 11 patients (9%), in which case we used clinical criteria to establish cognitive and neurodevelopmental progress. We classified seizures and epilepsy syndromes according to the ILAE criteria.^1^, ^14^, ^15^, ^16^ We used the definitions “early‐onset,” when absence seizures onset occurred before 3 years of age, and “drug resistance,” as failure of adequate trials of two tolerated, appropriately chosen and used antiseizure medications (ASMs) to achieve sustained seizure freedom.^17^

Genetic analysis was conducted through NGS epilepsy gene panels or whole‐exome sequencing. We collected data on each genetic variant, including the gene, transcript, variant type, inheritance pattern, family segregation, and the reported ACMG classification. Reported ACMG classifications were verified for consistency using the VarSome Premium tool (www.varsome.com), which integrates updated information from population allele frequency databases (e.g., gnomAD v4.1), disease‐related variant databases (e.g., ClinVar and LOVD), *in silico* prediction tools (e.g., dbNSFP, REVEL, CADD, SIFT, etc.), and other datasets.

### 2.1 Statistical analysis

We used the Pearson *χ* ^2^ or Fisher exact tests, as appropriate, for binary or categorical variables, and the *t* test for continuous variables, to analyze univariate associations. The outcome variable was intellectual disability or other neurodevelopmental disorder at last follow‐up. To identify significant independent outcome predictors, we constructed a multivariable logistic regression model including the variables that emerged as significant (*p* < .05) in the univariate analyses, using a backward‐stepwise approach. We estimated odds ratios (ORs) and 95% CIs. Variables with high collinearity (variance inflation factor >5) were excluded from the multivariable model. No variable had more than 5% missing data: missing data were therefore omitted, no other correction or interpolation was undertaken. We set the threshold for statistical significance at *p* < .05. We performed data analysis using the StataNow/MP 18.5 statistical package.

## 3 RESULTS

### 3.1 Patients and genetic etiologies

We included 160 patients (111 female, 49 male) with a confirmed genetic etiology and EEG‐confirmed absence seizures.

**FIGURE 1:**
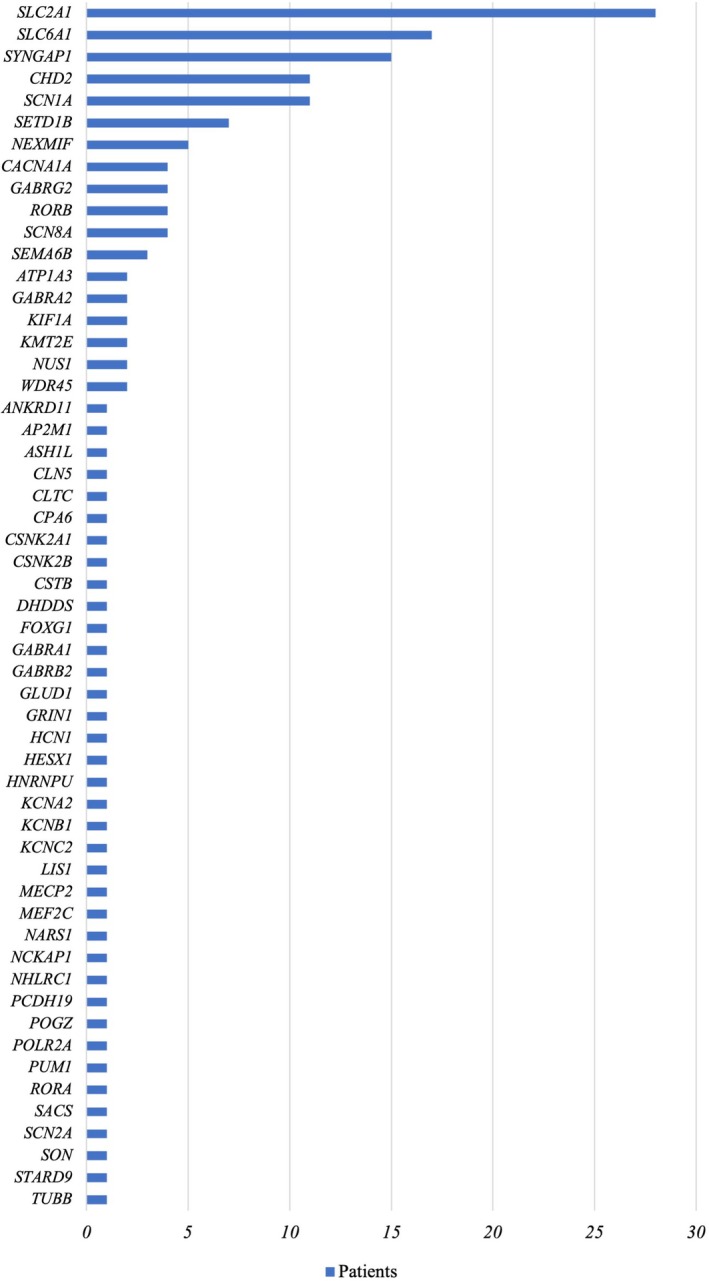
Genetic etiologies in decreasing frequency order in our cohort.

We found high genetic heterogeneity with 56 different genes involved; among these the most frequent were *SLC2A1* (*n* = 27), *SLC6A1* (*n* = 17), *SYNGAP1* (*n* = 15), *CHD2* (*n* = 11), *SCN1A* (*n* = 11), *SETD1B* (*n* = 7), *NEXMIF* (*n* = 5), *SCN8A* (*n* = 4), *GABRG2* (*n* = 4), *RORB* (*n* = 4), *CACNA1A* (*n* = 3), *SEMA6B* (*n* = 3), *ATP1A3* (*n* = 2), *GABRA2* (*n* = 2), *KIF1A* (*n* = 2), *KMT2E* (*n* = 2), and *NUS1* (*n* = 2). All other genetic etiologies accounted for one single individual each (Figure 1). Most variants were de novo (121/160, 76%), whereas 21 of 160 (13%) were heterozygous and inherited from an affected (*n* = 15) or unaffected (*n* = 6) parent, and 5 (3%) were compound heterozygous or homozygous. For the remaining 13 (8%) variants, the segregation status could not be determined. According to the ACMG classification, 111 (69%) were classified as pathogenic and 49 (31%) as likely pathogenic. One individual had two de novo pathogenic variants identified, one in *SLC2A1* and one in *CACNA1A*.

### 3.2 Seizure onset and semiology

The median age at any seizure onset was 2 years (range 0 months to 19 years), whereas the median age at absence seizures onset was 3 years (range 3 months to 21 years). Onset before 1 year of age occurred in 12 of 160 patients (7.5%), most commonly associated with *SLC2A1* (*n* = 7), but also with *CHD2* (*n* = 1), *MEF2C* (*n* = 1), *NEXMIF* (*n* = 1), and *SCN1A* (*n* = 1). Representative ictal EEG traces from children with absence onset before 1 year are provided in Figure S1. Follow‐up duration was highly variable, with age at last follow‐up ranging from 1 to 56 years (median 13 years). Most patients had experienced absence seizures at onset (107/160, 67%), whereas 25 (16%) had initially experienced febrile seizures, and 28 (18%) other seizure types. In 57 of 160 (35%) of patients, absence seizures were the only seizure type, whereas 104 of 160 (65%) had multiple seizure types.

In many patients (89/160, 56%), absence seizure semiology frequently extended beyond the typical features of impaired awareness, staring and interruption of activity, but included eyelid myoclonia in 67 (42%); other myoclonic components in 36 (23%); and oral, motor automatisms and/or autonomic components in 52 (33%). Ictal EEG recordings showed atypical features, that is, spike‐and‐wave complexes at less than 2.5 Hz, with irregular discharges, often asymmetrical,^18^ in 90 of 160 patients (56%). In the same recordings where absence seizures were captured, EEG background was normal in 105 of 160 (66%), whereas slowing was reported in 55 of 160 (34%). Interictal abnormalities were generalized (87/160, 54%), focal (14/160, 9%), or both (50/160, 31%), whereas they were not observed at all in 9 of 160 (6%).

Early onset of absences occurred in 94 of 160 (58%) of patients and was more frequently associated with absences with eyelid myoclonia (*p* = .004), abnormal EEG background (*p* = .011), and atypical ictal EEG features (*p* = .023).

Neuroimaging was unremarkable in most patients (144/160, 90%), whereas abnormal findings were reported in 6 of 160 (4%) including global cerebral atrophy (*CLN5*), lissencephaly (*LIS1*), myelination delay (*SETD1B*), white matter or vascular abnormalities (*SLC2A1*), and arachnoid cyst of the interpeduncular‐prepontine cistern (*SON*). Magnetic resonance imaging (MRI) was not performed in 10 of 160 (6%).

Precipitating factors for absences included hyperventilation, intermittent photic stimulation, and eye closure (Table 1; Figure S2).

**TABLE 1:** Clinical and EEG features of patients grouped by epilepsy syndromes.

Epilepsy syndrome	Genetic etiologies	Sex	Seizure type at onset	Early‐onset absences	Absence semiology	Precipitating factors	History of SE	DRE	Neurological examination	Other organ involvement	EEG background	EEG interictal discharges	EEG ictal patterns
CAE (5)	*CACNA1A, GABRG2, SCN1A, SLC2A1, SLC6A1*	M:2; F:3	Ab (4); FS (1)	0	Psycho‐motor arrest (4); +mild motor/automatic component (3)	HP (2); ILS (1)	0	0	Mild motor impairment (1); strabismus (1)	No	Normal (5)	Generalized (5)	Typical (5)
JAE (4)	*SETD1B* (2), *ASH1L, SLC2A1*	M:1; F:3	Ab (2); GTCS (2)	0	Psycho‐motor arrest (4); +mild motor component (1)	HP (1); ILS (1)	0	0	Normal (4)	No	Normal (4)	Generalized (4)	Typical (3); Atypical (1)
JME (2)	*GABRA2* (2)	F:2	FS (2)	0	Psycho‐motor arrest (2)	HP, ILS, eye closure (2)	0	0	Normal (2)	No	Normal (2)	Generalized (2)	Typical (2)
EEM (15)	*SYNGAP1* (4), *CHD2* (4), *CPA6, HCN1, KCNB1, KIF1A, RORB, SLC2A1, SLC6A1*	M:3; F:12	Ab (11); FS (2); Eyelid myoclonias (2)	7	Psycho‐motor arrest (4); +eyelid myoclonias (15); +myoclonias (1); +mild motor/automatic component (1)	HP (6); ILS (10); eye closure (7)	1	10	Ataxia (1); tremor (2); mild motor impairment (5); strabismus (2)	Ocular (1)	Normal (13); slow (2)	Generalized (10); focal (2); both (3)	Typical (11); atypical (4)
EMA (7)	*CHD2* (2), *SLC2A1* (2), *NEXMIF, RORA, SYNGAP1*	M:1; F:6	Ab (6); FS (1)	4	Psycho‐motor arrest (2); +eyelid myoclonias (2); +mild motor/automatic component (2); +myoclonias (7)	HP (3); ILS (3); eye closure (1)	1	6	Ataxia (2); mild motor impairment (3)	Genito‐urinary (1)	Normal (4); slow (3)	Generalized (3); focal and generalized (4)	Typical (1); atypical (6)
GEFS+ (1)	*SCN1A*	M	FS	1	Psycho‐motor arrest (1)	None	0	0	Normal	No	Normal	Generalized	Atypical
EMAtS (17)	*CHD2* (4), *SLC6A1* (3), *SLC2A1* (2), *ANKRD11, CACNA1A, GRIN1, KCNA2, KMT2E POLR2A, POGZ, RORB, WDR45*	M:5; F:12	Ab (9); FS (2); GTCS (1); MAt (4); Eyelid myoclonias (1)	13	Psycho‐motor arrest (7); +eyelid myoclonia (6); +myoclonia (3); +mild motor/automatic component (9)	HP (5); ILS (8); eye closure (2)	4	13	Ataxia (2); tremor (1); mild motor impairment (7); strabismus (2)	Cardiac (2); endocrinological (1); ocular (1); orthopedic (1)	Normal (10); slow (7)	Generalized (6); focal (1); both (10)	Typical (5); atypical (10); both (2)
DS (6)	*SCN1A* (6)	M:2; F:4	FS (4); GTCS (1); HS (1)	4	Psycho‐motor arrest (1); +eyelid myoclonias (1); +myoclonias (3); +mild motor/automatic component (2)	ILS (4); eye closure (1)	2	6	Ataxia (1); mild motor impairment (3); strabismus (1)	Hematological (1)	Normal (3); slow (3)	Generalized (1); focal (1); both (4)	Typical (1); atypical (5)
PME (5)	*SEMA6B* (2), *CSTB, NHLRC1, SACS*	M:1; F:4	Ab (3); FS (1); GTCS (1)	2	Psycho‐motor arrest (1); +eyelid myoclonias (1); +myoclonias (2); +mild motor/automatic component (2)	ILS (4); HP (1); eye closure (1)	1	4	Ataxia (4); myoclonus (3); mild motor impairment (2); pyramidal signs (1)	No	Slow (5)	Generalized (4); focal and generalized (1)	Atypical (5)
NC (98)	*SLC2A1* (21), *SLC6A1* (12), *SYNGAP1* (10), *SETD1B* (5), *NEXMIF* (4), *SCN1A* (4), *GABRG2* (3), *SCN8A* (3), *CACNA1A* (2), *MEF2C* (2), *RORB* (2), *NUS1* (2), *AP2M1, ATP1A3, CHD2, CLN5, CLTC, CSNK2A1, CSNK2B, DHDDS, FOXG1, GABRA1, GABRB2, GLUD1, HNRNPU, KCNC2, KIF1A, KMT2E, LIS1, MECP2, MEF2C, NARS1, NCKAP1, PCDH19, PUM1, SCN2A, SEMA6B, SON, STARD9, TUBB, WDR45*	M:33; F:65	Ab (71); FS (11); GTCS (7); focal to BTCS (1); Eyelid myoclonias (2); MS (5); ES (1)	59	Psycho‐motor arrest (46); +eyelid myoclonias (40); +mild motor/automatic component (34); +myoclonias (19)	HP (33); ILS (28); eye closure (13)	15	45	Microcephaly (5); macrocephaly (2); ataxia (17); myoclonus (2); tremor (7); movement disorder NOS (10); mild motor impairment (25); strabismus (7); pyramidal signs (1); extrapyramidal signs (2); hypertonus (1); hypotonia (1); nystagmus (2)	Hematological (2); gastroenterological (4); cardiac (2); ocular (2); PNS (1); metabolic (1); genito‐urinary (1)	Normal (63); slow (35)	Generalized (52); focal (9); both (28); normal (8)	Typical (41); atypical (56); both (1)

*Note*: Each row represents a syndrome, and each column lists a clinical or EEG feature, often with the number of patients (in parentheses) for each value.

Abbreviations: Ab, absences; BTCS, bilateral tonic–clonic seizures; CAE, childhood absence epilepsy; DRE, drug‐resistant epilepsy; DS, Dravet syndrome; EEM, epilepsy with eyelid myoclonia; EMA, epilepsy with myoclonic absence; EMAtS, epilepsy with myoclonic–atonic seizures; F, female; FS, febrile seizures; GEFS+, genetic epilepsy with febrile seizures plus; Glut1DS, glucose transporter‐1 deficiency syndrome; GTCS, generalized tonic–clonic seizures; HS, hemiclonic seizures; HP, hyperpnea; ILS, intermittent light stimulation; JAE, juvenile absence epilepsy; JME, juvenile myoclonic epilepsy; M, male; m, months; MAt, myoclonic–atonic seizures; MS, myoclonic seizures; NC, not classifiable; NOS, not otherwise specified; PME, progressive myoclonic epilepsy; PNS, peripheral nervous system; SE, status epilepticus; ES, Epileptic spasms; y, years.

In 24 of 160 (15%) patients, there was a history of convulsive status epilepticus (*n* = 2, including the following etiologies: *NHLRC1* and *SCN1A*), non‐convulsive status epilepticus (*n* = 21, *AP2M1, ATP1A3, CHD2, GABRG2, KCNA2, KMT2E, MECP2, NEXMIF, RORB, SCN1A, SCN8A, SEMA6B, SLC2A1, SLC6A1, STARD9, SYNGAP1*) or both (*n* = 1, *POGZ*) (Figure S3).

### 3.3 Other clinical features

**FIGURE 2:**
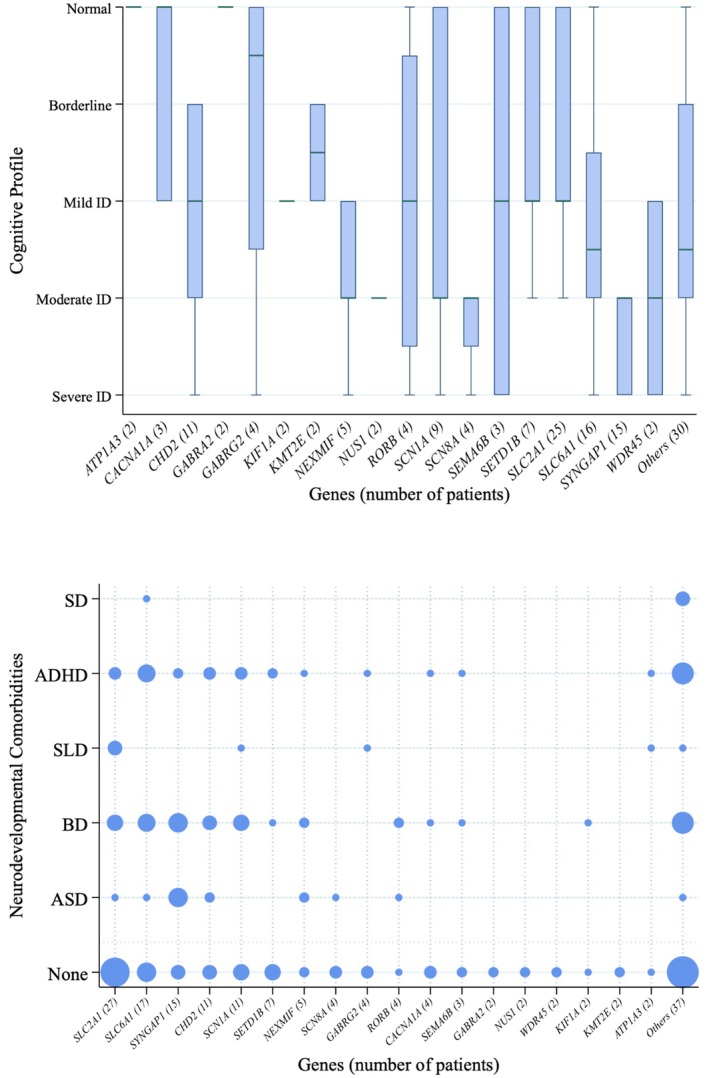
Intellectual disability and other neurodevelopmental comorbidities across genetic etiologies.

Developmental milestones were delayed in 87 of 160 patients (54%) including global (38%), only language (16%), or motor (1%) delay. When cognitive assessment had been performed (*n* = 146/160, 91%), cognitive function was normal in 32 of 146 (22%), borderline in 20 of 146 (14%), mildly impaired in 29 of 146 (20%), moderately impaired in 46 of 146 (32%), and severely impaired in 19 of 146 (13%). Other neurodevelopmental disorders were diagnosed in 78 of 160 patients (49%) including autism spectrum disorder, behavioral disorders, attention‐deficit/hyperactivity disorder (ADHD), and speech disorders (Figure 2). Anxiety (3%) and mood disorder (1%) were also reported.

Six of 160 patients (4%) had microcephaly and two of 160 (1%) macrocephaly at last follow‐up.

Facial dysmorphisms were observed in 23 of 160 patients (14%), ataxia in 27 of 160 (17%), and other movement disorders including tremor in 10 of 160 (6%) and paroxysmal dyskinesia in 4 of 160 (3%). Additional neurological abnormalities were observed in 66 of 160 (41%) such as mild motor impairment, strabismus, pyramidal or extrapyramidal signs, and abnormal muscle tone. Extra‐neurological comorbidities were present in 21 of 160 patients (13%) including hematological, gastroenterological, cardiac, ophthalmological, metabolic, endocrinological, urological, and orthopedic.

### 3.4 Syndrome classification

**FIGURE 3:**
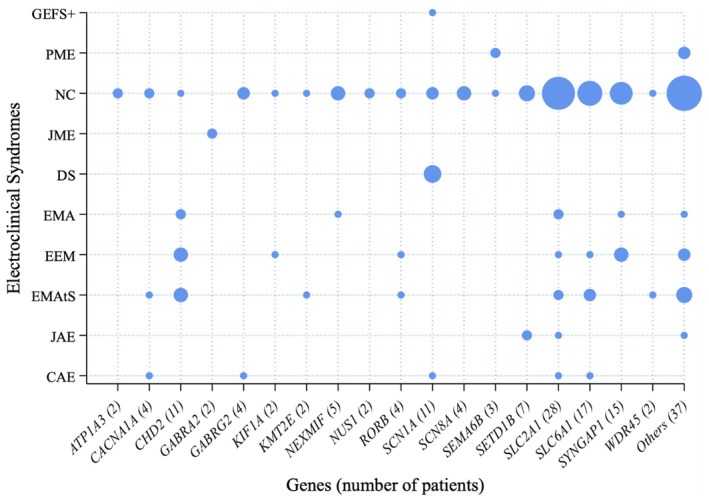
Electroclinical syndromes across genetic etiologies.

Most patients (106/160, 67%) were classified as having DEEs or epileptic encephalopathy (EE). Sixty‐four patients (40%) met the criteria for defined epilepsy syndromes, including epilepsy with myoclonic–atonic seizures (*n* = 17, 11%), epilepsy with eyelid myoclonia (*n* = 15, 9%), epilepsy with myoclonic absences (*n* = 7, 4%), childhood absence epilepsy (*n* = 7, 4%), Dravet syndrome (*n* = 6, 4%), progressive myoclonic epilepsy (*n* = 5, 3%), juvenile absence epilepsy (*n* = 4, 3%), and juvenile myoclonic epilepsy (*n* = 2, 1%), genetic epilepsy with febrile seizures plus (GEFS+) (*n* = 1, 1%). However, a significant proportion of patients (*n* = 96, 60%) could not be classified within the current ILAE classification framework (Figure 3). Early absence onset was common in epilepsy with myoclonic–atonic seizures (59%), epilepsy with myoclonic absences (67%), and GEFS+ (100%) (*p* = .005).

### 3.5 Treatment response

Most patients (*n* = 86, 54%) had drug‐resistant epilepsy^17^ with ongoing absence or other seizures at last follow‐up. The following treatments were reported as effective in reducing absence frequency: valproate (effective in 102/142, 72%), ethosuximide (in 72/106, 68%), benzodiazepines (in 40/82, 49%), lamotrigine (in 26/53, 49%), ketogenic diet (in 13/23, 57%), stiripentol (in association with clobazam; effective in 12/25, 80%), steroids (in 6/15, 40%), fenfluramine (in 5/7, 71%), levetiracetam (in 5/63, 8%), phenobarbital (in 4/13, 31%), zonisamide (in 4/13, 31%) rufinamide (in 4/11, 36%), cannabidiol (in 2/8, 25%), carbamazepine (in 2/14, 14%), topiramate (in 2/11, 18%), and cenobamate (in 1/1) (see Table 2). We note that these medications were often prescribed to address the full epilepsy syndrome rather than absence seizures in isolation. Therefore, percentages of effectiveness may reflect a reduction in overall seizure burden and not necessarily specific efficacy for absences. We found no difference in the prevalence of drug‐resistant epilepsy across the various etiologies. We confirmed the efficacy of specific treatments for absence seizures in certain genetic etiologies such as ketogenic diet in glucose transporter type 1 (GLUT1) deficiency syndrome and stiripentol and fenfluramine in *SCN1A*‐related epilepsies (Table 2).

**TABLE 2:** Genetic etiology and treatment efficacy.

	DRE (%)	BDZ	CBD	CBZ	CNB	ETS	FFA	HCT	KD	LCS	LEV	LTG	PB	PHT	RUF	STP	TPM	VPA	ZNS
ATP1A3 (*n* = 2)	100%	1 (0%)	–	–	–	2 (50%)	–	–	–	–	–	–	–	–	–	–	–	2 (50%)	–
CACNA1A (*n* = 4)	50%	1 (0%)	–	–	–	4 (25%)	–	–	–	–	–	3 (67%)	–	–	–	–	–	4 (75%)	–
CHD2 (*n* = 11)	91%	9 (44%)	–	1 (0%)	–	5 (60%)	1 (0%)	–	2 (0%)	–	7 (0%)	5 (40%)	2 (0%)	1 (0%)	–	–	2 (0%)	11 (55%)	2 (50%)
GABRA2 (*n* = 2)	0%	2 (100%)	–	–	–	2 (100%)	–	–	–	–	–	–	–	–	–	–	–	2 (100%)	–
GABRG2 (*n* = 4)	50%	2 (50%)	–	–	–	2 (100%)	–	–	–	–	1 (0%)	1 (100%)	–	–	–	–	1 (0%)	3 (67%)	–
KIF1A (*n* = 2)	0%	–	–	–	–	1 (0%)	–	–	–	–	–	–	–	–	–	–	–	2 (100%)	–
KMT2E (*n* = 2)	50%	1 (100%)	–	–	–	2 (100%)	–	1 (100%)	–	–	–	–	–	–	1 (100%)	1 (100%)	–	1 (100%)	–
NEXMIF (*n* = 5)	80%	5 (40%)	–	2 (0%)	–	4 (0%)	–	–	–	1 (0%)	5 (0%)	3 (33%)	–	–	1 (100%)	1 (100%)	–	4 (100%)	–
NUS1 (*n* = 2)	0%	–	–	–	–	–	–	–	–	–	2 (0%)	–	–	–	–	–	–	2 (100%)	–
RORB (*n* = 4)	50%	3 (0%)	–	1 (0%)	–	3 (33%)	–	2 (0%)	–	–	1 (0%)	2 (0%)	–	–	–	1 (0%)	1 (0%)	4 (50%)	1 (0%)
SCN1A (*n* = 11)	55%	6 (83%)	2 (0%)	3 (0%)	–	9 (78%)	5 (80%)	2 (0%)	3 (0%)	–	2 (0%)	2 (50%)	1 (0%)	–	–	5 (100%)	3 (0%)	10 (80%)	3 (33%)
SCN8A (*n* = 4)	75%	1 (0%)	–	1 (100%)	–	2 (100%)	–	–	–	1 (0%)	2 (50%)	1 (0%)	1 (0%)	–	–	–	1 (100%)	3 (0%)	–
SEMA6B (*n* = 3)	67%	2 (50%)	–	–	–	1 (100%)	–	1 (100%)	–	–	1 (0%)	1 (100%)	1 (100%)	–	–	1 (100%)	1 (0%)	2 (100%)	–
SETD1B (*n* = 7)	29%	3 (0%)	–	–	–	5 (80%)	–	–	–	–	2 (50%)	2 (100%)	–	–	–	–	–	6 (83%)	–
SLC2A1 (*n* = 28)	36%	11 (54%)	–	2 (0%)	–	17 (88%)	–	–	16 (75%)	–	9 (0%)	10 (60%)	2 (0%)	–	–	–	2 (0%)	24 (79%)	–
SLC6A1 (*n* = 17)	53%	8 (50%)	–	2 (0%)	–	11 (73%)	–	3 (33%)	1 (0%)	0	4 (50%)	8 (37%)	–	–	2 (0%)	1 (0%)	2 (0%)	16 (87%)	1 (100%)
SYNGAP1 (*n* = 15)	60%	7 (71%)	1 (100%)	–	–	9 (55%)	–	1 (0%)	–	–	7 (0%)	6 (50%)	–	–	1 (0%)	2 (100%)	–	14 (57%)	–
WDR45 (*n* = 2)	100%	1 (100%)	–	–	–	1 (100%)	–	–	–	–	1 (0%)	–	–	–	–	–	1 (100%)	1 (100%)	–
Others (*n* = 37)	49%	20 (40%)	5 (20%)	3 (33%)	1 (100%)	29 (65%)	–	5 (60%)	1 (0%)	3 (0%)	19 (5%)	9 (44%)	6 (50%)	–	5 (20%)	2 (50%)	7 (0%)	34 (68%)	6 (17%)

*Note*: Each row represents a specific genetic etiology. The first column shows the proportion of patients with drug‐resistant epilepsy. The remaining columns indicate the number of patients who tried each treatment, with the percentage of those where treatment was effective shown in parentheses.

Abbreviations: ASMs, antiseizure medications; BDZ, benzodiazepine; CBD, cannabidiol; CBZ, carbamazepine; CNB, cenobamate; ETS, ethosuximide; DR, drug resistance; FFA, fenfluramine; HCT, hydrocortisone; KD, ketogenic diet; LCS, lacosamide; LEV, levetiracetam; LTG, lamotrigine; n, number; NA, not available; PB, phenobarbital; PHT, phenytoin; RUF, rufinamide; STP, stiripentol; TPM, topiramate; VPA, valproate; ZNS, zonisamide.

### 3.6 Genotype–phenotype correlations

Early‐onset absences were observed in 94 (58%), and more frequently associated with *SLC2A1* (*n* = 12), *SYNGAP1* (*n* = 10), *SLC6A1* (*n* = 8), *SCN1A* (*n* = 6), *CHD2* (*n* = 4), *NEXMIF* (*n* = 4), *GABRG2* (*n* = 2), *KIF1A* (*n* = 2), *KMT2E* (*n* = 2), and *RORB* (*n* = 2). Among early‐onset seizures, the majority were classified as atypical in 44 of 67 (65%), whereas 21 of 67 patients (31%) had typical absences, and 2 of 67 (3%) had both types.

Atypical absences were more common with *SLC2A1* (*n* = 15), *SYNGAP1* (*n* = 9), *CHD2* (*n* = 8), *SCN1A* (*n* = 8), *NEXMIF* (*n* = 5), *SLC6A1* (*n* = 5), *SCN8A* (*n* = 3), *SEMA6B* (*n* = 3), *KMT2E* (*n* = 2), and *RORB* (*n* = 2).

Among absence precipitating factors, hyperventilation was common with variants in *CACNA1A, GABRA2, GABRG2, SETD1B, SLC2A1, SLC6A1*, and *SYNGAP1*; intermittent photic stimulation in *CACNA1A, CHD2, GABRA2, KMT2E, RORB, SCN1A, SEMA6B, SETD1B*, and *SYNGAP1*; and eye closure in *CHD2, GABRA2, NEXMIF*, and *SYNGAP1*.

We observed genetic heterogeneity across the various epilepsy syndromes, except obviously for Dravet syndrome. Epilepsy with myoclonic–atonic seizures was most frequently (i.e., more than one patient) associated with pathogenic variants in *CHD2, SLC2A1*, and *SLC6A1*: epilepsy with eyelid myoclonia in *CHD2* and *SYNGAP1*: epilepsy with myoclonic absences in *CHD2* and *SLC2A1*: progressive myoclonic epilepsy in *SEMA6B:* JAE in *SETD1B*, and JME in *GABRA2* (Table 1, Figure 3).

A minority of patients (*n* = 25, 16%) did not have intellectual disability or other neurodevelopmental comorbidities, including patients with *SLC2A1* (*n* = 4), *SCN1A* (*n* = 3), *GABRA2* (*n* = 2), *GABRG2* (*n* = 2), and *SETD1B* (*n* = 2) pathogenic variants (Figure 2). These patients with favorable neurodevelopmental outcomes had higher age at seizure onset (mean 56 vs 38 months, *p* = .039). We found significant univariate associations between intellectual disability and other neurodevelopmental comorbidities and early absence onset (*p* = .002), absences associated with myoclonic components (*p* = .045), drug resistance (*p* = .003), slow EEG background (*p* = .017), early developmental delay (*p* < .001), and abnormal neurological examination (*p* ≤ .001). Syndromic classification was also associated with neurodevelopmental outcomes, which were worse in epilepsy with myoclonic–atonic seizures, epilepsy with eyelid myoclonia, epilepsy with myoclonic absences, CAE, Dravet syndrome, progressive myoclonic epilepsy, and unclassified syndromes, whereas better outcomes were linked to JAE, JME, and GEFS+ (*p* < .001). With logistic multivariate regression analysis, we confirmed early developmental delay, drug‐resistant epilepsy, and early absence onset as independent predictors of unfavorable outcomes (Table 3).

**TABLE 3:** Multivariable logistic regression model analyzing the association with unfavorable neurodevelopmental outcome as outcome variable.

Variable	Odds ratio	*Z*	*p*‐value	95% confidence interval
Drug‐resistance	5.6	3.08	.002	1.878–16.960
Early absence onset	3.3	2.29	.022	1.191–9.331
Developmental delay	1.6	2.33	.020	1.073–2.251
Constant	.84	−.44	.657	.388–1.819

*Note*: Pseudo *R* ^2^ = .2228.

### 3.7 Novel genes associated with absence seizures

We identified four genes—*HESX1*, *NCKAP1*, *SON*, and *STARD9*—not previously associated with absence seizures, including one (*STARD9*) with limited evidence for epilepsy and not yet included in the Online Mendelian Inheritance in Man (OMIM).

*HESX1* is a member of the paired‐like class of homeobox genes, essential for early differentiation of the forebrain and adenohypophysis. The first homozygous *HESX1* pathogenic variant in humans (R160C) was described in two siblings of consanguineous background with septo‐optic dysplasia.^19^ Subsequently, different autosomal dominant and recessive variants have been described in this gene, with phenotypes ranging from isolated growth hormone deficiency to combined pituitary hormone deficiencies and septo‐optic dysplasia without clear genotype–phenotype correlation. Generally, heterozygous variants produce milder phenotypes, with isolated growth hormone deficiency and an undescended posterior pituitary, although some forebrain abnormalities have been reported. Penetrance is highly variable.^20^ Our patient had a heterozygous frameshift *HESX1* variant (NM_003865.2:c.305_306delAG; p.Glu102Valfs*5), inherited from a healthy father. The variant is reported in 6 of 1 613 664 alleles (with no homozygotes) in the gnomAD database (v4.1.0). The variant is predicted to undergo nonsense‐mediated RNA decay (NMD) and is classified as likely pathogenic (ACMG criteria: PVS1, PM2).

The patient began experiencing absence seizures at 26 months, initially not controlled with ethosuximide, but is now (age 7 years) seizure‐free on valproate and clonazepam, has mild intellectual disability, and normal brain MRI. Epilepsy has been reported in septo‐optic‐pituitary dysplasia,^21^ but a specific association with absence seizure is novel.

The *NCKAP1* gene (also known as *NAP1*) encodes for a protein that regulates neuronal cytoskeletal dynamics and is essential for neuronal differentiation in the developing brain. Heterozygous de novo and inherited disruptive rare variants in *NCKAP1* have been associated with neurodevelopmental disorders, with a core autism spectrum disorder phenotype. Additional features include language and motor delay, and variable degrees of intellectual disability. Seizures or epilepsy have been reported in only three individuals without further details.^22^ Our patient has a de novo heterozygous missense *NCKAP1 v*ariant (NM_205842.3:c.2591A>G; p.His864Arg). The variant is reported in 1 of 1 457 648 alleles (with no homozygotes) in the gnomAD database (v4.1.0) and involves an amino‐acidic residue located in a position highly intolerant to missense substitutions according to Metadome (https://stuart.radboudumc.nl/metadome). The c.2591A>G variant results in the p.His864Arg amino‐acidic substitution predicted to be damaging and classified as likely pathogenic (ACMG criteria: PS2, PM2, PP3). Our patient had absence onset at 17 months, followed by generalized tonic–clonic seizures. Borderline cognitive function and a behavioral disorder were present at last follow‐up at age 3 years. He is seizure‐free on valproate, ethosuximide, and clonazepam polytherapy.

The *SON* gene encodes for a key component of the spliceosomal complex and a critical mediator of constitutive and alternative splicing. De novo *SON* pathogenic variants cause a severe multisystem disorder (Zhu‐Tokita‐Takenouchi‐Kim syndrome), with developmental delay, persistent feeding difficulties, and congenital malformations, also including abnormal gyral pattern, ventriculomegaly, Arnold–Chiari malformation, arachnoid cysts, loss of periventricular white matter, and corpus callosum and cerebellar hypoplasia.^23^ Epilepsy or febrile and afebrile seizures have been reported in about 50% of affected individuals, with onset between 1 and 6 years, but no further details are available.^24^, ^25^ Our patient had a heterozygous *SON* in‐frame deletion variant (NM_032195.3:c.3153_3179del; p.Pro1052_Ser1060del). The variant causes an in‐frame deletion of 9 amino acids in a *SON* non‐repeat region that is intolerant to variations according to Metadome (https://stuart.radboudumc.nl/metadome/). The variant is also present in the patient's sister, presenting with mild intellectual disability and scoliosis. Parental DNA was unavailable because both sisters were adopted. The variant is reported in 54 of 1 613 166 alleles (with no homozygotes) in the gnomAD database (v4.1.0). We classified the variant as likely pathogenic (ACMG criteria: PM2, PM4, PP4). Our patient began experiencing febrile and afebrile convulsive seizures at 30 months, and atypical absences at 36 months. Her brain MRI (age 9 years) showed an arachnoid cyst of the interpeduncular‐prepontine cistern, causing splaying of the cerebral peduncles and oculomotor nerves. At last follow‐up at age 10 years, she had borderline cognitive function, ADHD, and was seizure‐free on ethosuximide and lamotrigine. She has mild scoliosis and congenital bilateral esotropia, which was surgically corrected.

The *STARD9* gene encodes for the StAR‐related lipid transfer domain‐containing protein 9, also known as KIF16A‐related kinesin‐like protein, and belonging to the kinesin superfamily proteins (Torres et al., 2011). This protein is conserved in vertebrates and contains a kinesin motor domain, the forkhead‐associated (FHA) domain, and the StAR‐related lipid‐transfer (START) domain. *STARD9* is ubiquitously expressed in almost all tissues including skin, heart, and brain. *STARD9* is not yet mentioned in the OMIM database, but a patient with severe intellectual disability, epilepsy, acquired microcephaly, and blindness was reported with a homozygous frameshift pathogenic variant in this gene.^26^ Her seizures occurred on awakening, with occasional distal jerking and were controlled on medication. Brain MRI was normal. Depletion of STARD9 or overexpression of C‐terminally truncated *STARD9* mutants induce spindle assembly defects in cultured human cells^27^; accordingly, mitotic defects were found in the patient's lymphoblast cells, including multipolar spindle formation, pericentriolar material fragmentation, and centrosome amplification.^26^ Our patient is compound heterozygous for two missense variants (NM_020759.3:c.2080C>T; p.Leu694Phe & NM_020759.3:c.10225A>G; p.Met3409Val). The *STARD9* gene is inferred to follow a recessive pattern of inheritance according to the DOMINO tool (https://domino.iob.ch/). The c.2080C>T variant, inherited from the mother, is reported in 5 of 1 384 492 alleles (with no homozygotes), whereas the c.10225A>G variant, inherited from the father, is reported in 117 of 1 384 946 alleles (with no homozygotes) in the gnomAD database (v4.1.0). Given that *STARD9* is not currently established as a disease‐associated gene, it is considered a gene of uncertain significance (GUS). Consequently, ACMG variant classification is not applicable to the identified variants. Our patient experienced febrile seizures at age 14 months, followed by atypical absence seizures at 36 months, including absences with eyelid myoclonia and negative myoclonus. Her EEG was consistent with EE with spike–wave activation in sleep (EE‐SWAS), which improved following hydrocortisone treatment. Her seizures were then controlled with ethosuximide and valproate. Developmental progress was not significantly delayed and head circumference was normal, but minor facial dysmorphisms were present. EEG showed a photoparoxysmal response. Brain MRI was normal. At last follow‐up at age 9 years, the girl was seizure‐free and off antiseizure treatment, and had borderline intellectual function, ADHD, and expressive language difficulties.

## 4 DISCUSSION

This study of 160 patients with genetically confirmed epilepsy with absence seizures highlights the heterogeneous clinical and genetic context in which absence seizures may appear, and how relevant a broad phenotypic recognition is in guiding genetic testing.

Although absence seizures are classically associated with IGEs, particularly CAE, JAE, and JME, our findings demonstrate that they frequently occur in the context of multiple monogenic conditions, with over two‐thirds of our cohort falling within the DEE/EE spectrum. The semiology of absence seizures often extended beyond the typical brief behavioral arrest and included features such as eyelid myoclonia, other myoclonic components, and automatisms. Atypical EEG features, including background slowing and irregular spike‐and‐wave discharges, were also common, diverging from the classical 3 Hz generalized spike‐and‐wave ictal pattern of IGEs. These findings emphasize the need for clinicians to maintain a high index of suspicion for monogenic etiologies when absence seizures present with early onset, atypical clinical, and/or EEG features, and when associated with developmental delay or drug resistance.

Insights from genetic animal models of absence epilepsy, such as genetic absence epilepsy rat from Strasbourg (GAERS) and Wistar Albino Glaxo from Rijswijk (WAG/Rij) rats, support a major role of thalamo‐cortical circuitry in absence generation. These models demonstrate spontaneous spike–wave discharges with high predictive validity to human absence seizures.^28^ There is evidence that epileptiform abnormalities originate within the thalamo‐cortical system, with seizure initiation in the deep layers of the somatosensory cortex (S1), and subsequent rapid generalization via cortico‐thalamic and intracortical pathways.^29^ Our findings indicate that a wide range of genetic alterations can converge on a common pathophysiological mechanism involving this network, which would underlie the shared absence seizure phenotype across different genetic disorders. Despite genetic diversity, convergence of absence seizures toward a relatively consistent clinical and EEG phenotype suggests that diverse molecular disruptions may all impair the thalamo‐cortical network during development, and that absence seizures in childhood may represent a final common age‐dependent expression of multiple distinct pathogenic mechanisms. Neuroimaging was unremarkable in the majority of our cohort, suggesting that such impairment is not caused by structural abnormalities in most cases. Absence seizures occur predominantly in childhood, with earlier onset often observed in monogenic etiologies and later onset in presumed oligogenic or polygenic forms. In our monogenic cohort, we observed a high proportion with drug‐resistant epilepsy, including the persistence of seizures into adulthood.

We identified 56 distinct monogenic etiologies, with *SLC2A1, SYNGAP1*, *SLC6A1, SCN1A*, *CHD2*, and *NEXMIF* being the most frequently implicated genes (*n* > 4). However, many other genes were rare or unique. We identified four genes—*HESX1, NCKAP1, SON*, and *STARD9*—not previously associated with absence seizures, one of which (*STARD9*) is not currently included in the OMIM database. These novel gene associations expand the known genetic architecture of absence epilepsy and further support the concept that absence seizures can emerge from diverse molecular mechanisms converging on shared neurodevelopmental pathways. Inclusion of these novel associations highlights the value of comprehensive genetic screening and phenotype‐driven variant interpretation, particularly when clinical features are atypical or syndromic.

Over half of the patients could not be classified within the current ILAE syndrome framework,^14^ illustrating the limitations of existing classification systems, or any effort to define a syndromic framework, in the context of genetic epilepsy. These limitations underscore the need for evolving classification models that incorporate genetic data alongside traditional electroclinical features, especially as NGS becomes increasingly used in clinical practice. Our cohort includes a continuum of epilepsy phenotypes, ranging from classical IGEs syndromes to the most varied DEE/EE spectrum. Yet, they could all still be classified under the controversial umbrella of GGE.^1^ A large genome‐wide association study identified 19 “GGE” loci^30^ with a strong contribution from common genetic variation. When analyzing individual syndromes, that study found that up to 90% of liability is attributable to common variants in the JAE subtype, making it among the highest of over 700 traits reported in a large genome‐wide association study (GWAS) atlas.^31^ These findings suggest that absence seizures exist along a continuum from monogenic to polygenic etiologies, further challenging strict dichotomies in current classifications.

The concept of a rigid, “pure IGE” group risks overlooking its underlying biological biodiversity, thereby hindering our understanding of the full clinical spectrum, pathophysiologic mechanism, and potential therapeutic approaches.^32^ We acknowledge that, considering the population of patients with absence seizures at large, our cohort is likely biased toward individuals with more severe phenotypes, developmental comorbidities, and/or drug resistance, as genetic testing was typically pursued in these contexts. This ascertainment bias partly explains the relatively low representation of CAE and JAE in our study, and therefore the high proportion of DEE and poor developmental outcomes should not be generalized to all individuals with absence seizures. The Epi25 analysis of over 9000 epilepsy individuals and 8000 controls showed the strongest enrichment of ultra‐rare deleterious variants in DEEs, with more modest enrichment in GGE and the lowest in non‐acquired focal epilepsies, and no single gene reaching exome‐wide significance in GGE.^33^ These findings reinforce that although absence seizures can occur in monogenic epilepsies, such cases represent a minority of all GGEs.

Some well‐defined disorders such as Dravet syndrome, showed consistent genotype–phenotype alignment. However, other syndromes, such as epilepsy with myoclonic–atonic seizures and epilepsy with eyelid myoclonia, were genetically heterogeneous. In some instances, the clinical presentation may suggest a specific genetic etiology: for example, early‐onset absences, along with ataxia, epilepsy with myoclonic–atonic seizures, and exercise‐induced dyskinesia are characteristic of GLUT1 deficiency syndrome.^10^, ^34^, ^35^ Similarly, eyelid myoclonia, myoclonic–atonic seizures, eating‐induced reflex seizures, and photosensitivity in the context of a DEE may point toward pathogenic variants in *SYNGAP1*.^36^, ^37^ On the other hand, some electroclinical syndromes featuring absence seizures may be recognizable despite an unknown or heterogeneous genetic basis, for example, epilepsy with myoclonic–atonic seizures.^38^ Despite being classically associated with atypical absences, no patients with Lennox–Gastaut syndrome were included in our cohort, possibly reflecting cohort selection, referral patterns, and the low rate genetic diagnosis currently achieved in this condition.^15^, ^39^

A significant proportion of patients in our cohort exhibited neurodevelopmental comorbidities (84%) and drug‐resistant epilepsy (54%). Early developmental delay, syndromic classification, and drug resistance were identified as independent predictors of cognitive and developmental impairment, whereas absences seizures only vs multiple seizure types was not associated with outcomes. These findings provide clinicians with important early red flags that should prompt comprehensive developmental assessment and genetic testing. A small subset of patients (16%) had favorable neurodevelopmental outcomes, particularly those with later seizure onset. Even in CAE, considered to be a “benign” disorder usually outgrowing epilepsy by adolescence, unfavorable psychosocial outcomes have been reported when compared with another chronic medical illness or with self‐limited epilepsy with centrotemporal spikes (e.g., rates of high school dropout, unplanned pregnancy, psychiatric disorder, unskilled work, or unemployed).^40^, ^41^

Although traditional ASMs such as valproate and ethosuximide were commonly used and often effective in controlling absence seizures, more than half of the cohort remained drug‐resistant at last follow‐up, with ongoing absence and/or other seizures. Disease‐specific therapies—such as ketogenic diet in *SLC2A1*‐related epilepsies^34^ and stiripentol or fenfluramine in *SCN1A* ^42^, ^43^, ^44^—were effective in those select cases. This specificity highlights the potential of precision medicine approaches to improve outcomes in monogenic epilepsies and further supports early genetic testing, although treatment specific to the underlying pathophysiological mechanisms is still available only for a minority of genetic epilepsies.^45^

This study benefits from a large, multicentric design, systematic phenotyping, and strict inclusion criteria based on EEG‐recorded absences and confirmed monogenic diagnoses. However, it is limited by its retrospective nature, potential referral bias toward more severe phenotypes, and the limited longitudinal follow‐up for neurodevelopmental outcomes. Moreover, the exclusion of patients with variants of uncertain significance may have underestimated the contribution of some genes.

## 5 CONCLUSIONS

Our findings demonstrate that absence seizures are a frequent but clinically and genetically heterogeneous feature of monogenic epilepsies, often associated with broader neurological and developmental abnormalities. Early onset, atypical electroclinical semiology, developmental delay, and drug resistance should prompt consideration of a genetic diagnostic work‐up to facilitate early diagnosis, syndrome clarification, and the adoption of more appropriate or even precision treatment strategies.

## AUTHOR CONTRIBUTIONS

S.B. contributed to the study conceptualization, data analysis and interpretation, and drafting of the study. I.G., M.L.R., E.P., and D.M. contributed to data acquisition and analysis and interpretation. M.M., F.P., C.F., L.G., E.C., C.M., E.M.I., C.D.B., M.R., A.C., J.P., T.L.B., F.D., L.L., F.B., M.P., D.B., A.D.D., M.T., N.S., R.S., D.C., and L.C. contributed to data acquisition and critical revision of the manuscript. R.G. contributed to the study conceptualization, data interpretation, critical revision, and final approval.

## FUNDING INFORMATION

None.

## CONFLICT OF INTEREST STATEMENT

None of the authors has any conflict of interest to disclose in relation to the contents of this manuscript.

## ETHICS STATEMENT

We confirm that we have read the Journal's position on issues involved in ethical publication and affirm that this report is consistent with those guidelines.

## Supporting information


Figure S1.

Figure S2.

Figure S3.

## Data Availability

The data that support the findings of this study are available from the corresponding author upon reasonable request.

## APPENDIX A

LICE Collaborative Group

Carmen Barba (Neuroscience and Human Genetics Department, Meyer Children's Hospital IRCCS, Florence, Italy; Department of Neuroscience, Pharmacology and Child Health, University of Florence, Florence, Italy), Emanuele Bartolini (Department of Developmental Neuroscience, IRCCS Stella Maris Foundation, Pisa, Italy), Pia Bernardo (Department of Neurosciences, Pediatric Psychiatry and Neurology, Santobono‐Pausilipon Children's Hospital, A.O.R.N., Naples, Italy), Maria Paola Canevini (Child Neuropsychiatry Unit, Epilepsy Center, San Paolo Hospital, Department of Health Sciences, University of Milan, Milan, Italy), Gaetano Cantalupo (Child Neuropsychiatric Unit, Department of Engineering for Innovation Medicine, University of Verona, Verona, Italy), Susanna Casellato (Child Neuropsychiatry Unit, Department of Woman's and Child's Health, Center of Pediatric Epilepsies, Azienda Ospedaliera Universitaria, Sassari, University of Sassari, Sassari, Italy), Mara Cavallin (Neuroscience and Human Genetics Department, Meyer Children's Hospital IRCCS, Florence, Italy), Ilaria Contaldo (Pediatric Neurology and Psychiatry, Dipartimento della Salute Della Donna, del Bambino e di Sanità Pubblica, Fondazione Policlinico Universitario A. Gemelli, IRCCS, Rome), Alessandro Ferretti (Pediatrics Unit, Neuroscience, Mental Health and Sense Organs (NESMOS) Department, Faculty of Medicine and Psychology, Sapienza University of Rome, Rome), Maria Luigia Gambardella (Pediatric Neurology and Psychiatry, Dipartimento Della Salute della Donna, del Bambino e di Sanità Pubblica, Fondazione Policlinico Universitario A. Gemelli, IRCCS, Rome), Tiziana Granata (Department of Pediatric Neuroscience, Fondazione IRCCS Istituto Neurologico Carlo Besta, Milan, Italy), Giuliana Lentini (Department of Human Neuroscience‐ Sapienza University of Rome), Anna Luchetti (Child Neuropsychiatry Unit, S. Andrea Hospital, Rome, Italy), Federico Melani (Neuroscience and Human Genetics Department, Meyer Children's Hospital IRCCS, Florence, Italy), Pasquale Parisi (Pediatrics Unit, Neuroscience, Mental Health and Sense Organs (NESMOS) Department, Faculty of Medicine and Psychology, Sapienza University of Rome, Rome), Simona Pellacani (Neuroscience and Human Genetics Department, Meyer Children's Hospital IRCCS, Florence, Italy; Department of Neuroscience, Pharmacology and Child Health, University of Florence, Florence, Italy), Laura Pietrangelo (Neuroscience and Human Genetics Department, Meyer Children's Hospital IRCCS, Florence, Italy), Tiziana Pisano (Neuroscience and Human Genetics Department, Meyer Children's Hospital IRCCS, Florence, Italy), Ilaria Onida (Child Neuropsychiatry Unit, Department of Woman's and Child's Health, Center of Pediatric Epilepsies, Azienda Ospedaliera Universitaria, Sassari, University of Sassari, Sassari, Italy), Emilia Ricci (Child Neuropsychiatry Unit, Epilepsy Center, San Paolo Hospital, Department of Health Sciences, University of Milan, Milan, Italy), Aglaia Vignoli (Childhood and Adolescence Neurology and Psychiatry Unit, ASST GOM Niguarda, and Department of Health Sciences, Università Degli Studi di Milano, Milan, Italy).

## References

[1] Hirsch E , French J , Scheffer IE , Bogacz A , Alsaadi T , Sperling MR , et al. ILAE definition of the idiopathic generalized epilepsy syndromes: position statement by the ILAE task force on nosology and definitions. Epilepsia. 2022;63(6):1475–1499. 35503716 10.1111/epi.17236

[2] Jallon P , Latour P . Epidemiology of idiopathic generalized epilepsies. Epilepsia. 2005;46(Suppl 9):10–14. 10.1111/j.1528-1167.2005.00309.x 16302871

[3] Brigo F , Striano P , Belcastro V . A reappraisal of atypical absence seizures in children and adults: therapeutic implications. Expert Opin Pharmacother [Internet]. 2019 Nov 22 [cited 2025 Jun 12];20(17):2115–2120. Available from: https://www.tandfonline.com/doi/full/10.1080/14656566.2019.1656716 31446808 10.1080/14656566.2019.1656716

[4] Scheffer IE , Zuberi S , Mefford HC , Guerrini R , McTague A . Developmental and epileptic encephalopathies. Nat Rev Dis Primers [Internet]. 2024 Sep 5 [cited 2024 Oct 30];10(1):61. Available from: https://www.nature.com/articles/s41572‐024‐00546‐6 39237642 10.1038/s41572-024-00546-6

[5] Penry JK , Porter RJ , Dreifuss FE . Simultaneous recording of absence seizures with video tape and electroencephalography: a study of 374 seizures in 48 patients. Brain [Internet]. 1975 [cited 2025 Jun 2];98(3):427–440. Available from: https://academic.oup.com/brain/article‐lookup/doi/10.1093/brain/98.3.427 1182486 10.1093/brain/98.3.427

[6] Beniczky S , Trinka E , Wirrell E , Abdulla F , Al Baradie R , Alonso Vanegas M , et al. Updated classification of epileptic seizures: position paper of the international league against epilepsy. Epilepsia [Internet]. 2025 Apr 23 [cited 2025 Jun 11];66:1804–1823. 10.1111/epi.18338 40264351 PMC12169392

[7] Sadleir LG , Farrell K , Smith S , Connolly MB , Scheffer IE . Electroclinical features of absence seizures in childhood absence epilepsy. Neurology. 2006;67(3):413–418. 16894100 10.1212/01.wnl.0000228257.60184.82

[8] Guerrini R , Balestrini S , Wirrell EC , Walker MC . Monogenic epilepsies: disease mechanisms, clinical phenotypes, and targeted therapies. Neurology [Internet]. 2021 Oct 26 [cited 2022 Mar 27];97(17):817–831. 10.1212/WNL.0000000000012744 34493617 PMC10336826

[9] Oliver KL , Scheffer IE , Bennett MF , Grinton BE , Bahlo M , Berkovic SF . Genes4Epilepsy: an epilepsy gene resource. Epilepsia. 2023;64(5):1368–1375. 36808730 10.1111/epi.17547 PMC10952165

[10] Suls A , Mullen SA , Weber YG , Verhaert K , Ceulemans B , Guerrini R , et al. Early‐onset absence epilepsy caused by mutations in the glucose transporter GLUT1. Ann Neurol. 2009;66(3):415–419. 19798636 10.1002/ana.21724

[11] Arsov T , Mullen SA , Damiano JA , Lawrence KM , Huh LL , Nolan M , et al. Early onset absence epilepsy: 1 in 10 cases is caused by GLUT1 deficiency. Epilepsia. 2012;53(12):e204–e207. 23106342 10.1111/epi.12007

[12] Krey I , von Spiczak S , Johannesen KM , Hikel C , Kurlemann G , Muhle H , et al. L‐serine treatment is associated with improvements in behavior, EEG, and seizure frequency in individuals with GRIN‐related disorders due to null variants. Neurotherapeutics. 2022;19(1):334–341. 34997442 10.1007/s13311-021-01173-9 PMC9130352

[13] Richards S , Aziz N , Bale S , Bick D , Das S , Gastier‐Foster J , et al. Standards and guidelines for the interpretation of sequence variants: a joint consensus recommendation of the American College of Medical Genetics and Genomics and the Association for Molecular Pathology. Genet Med. 2015;17(5):405–424. 25741868 10.1038/gim.2015.30 PMC4544753

[14] Scheffer IE , Berkovic S , Capovilla G , Connolly MB , French J , Guilhoto L , et al. ILAE classification of the epilepsies: position paper of the ILAE Commission for Classification and Terminology. Epilepsia [Internet]. 2017 Apr [cited 2020 Dec 23];58(4):512–521. 10.1111/epi.13709 28276062 PMC5386840

[15] Specchio N , Wirrell EC , Scheffer IE , Nabbout R , Riney K , Samia P , et al. International league against epilepsy classification and definition of epilepsy syndromes with onset in childhood: position paper by the ILAE task force on nosology and definitions. Epilepsia. 2022;63(6):1398–1442. 35503717 10.1111/epi.17241

[16] Zuberi SM , Wirrell E , Yozawitz E , Wilmshurst JM , Specchio N , Riney K , et al. ILAE classification and definition of epilepsy syndromes with onset in neonates and infants: position statement by the ILAE task force on nosology and definitions. Epilepsia. 2022;63(6):1349–1397. 35503712 10.1111/epi.17239

[17] Kwan P , Arzimanoglou A , Berg AT , Brodie MJ , Allen Hauser W , Mathern G , et al. Definition of drug resistant epilepsy: consensus proposal by the ad hoc task force of the ILAE commission on therapeutic strategies. Epilepsia [Internet]. 2010 Jun [cited 2023 Nov 7];51(6):1069–1077. 10.1111/j.1528-1167.2009.02397.x 19889013

[18] Panayiotopoulos CP . Typical absence seizures and related epileptic syndromes: assessment of current state and directions for future research. Epilepsia. 2008;49(12):2131–2139. 19049569 10.1111/j.1528-1167.2008.01777.x

[19] Dattani MT , Martinez‐Barbera JP , Thomas PQ , Brickman JM , Gupta R , Mårtensson IL , et al. Mutations in the homeobox gene HESX1/Hesx1 associated with septo‐optic dysplasia in human and mouse. Nat Genet [Internet]. 1998 Jun [cited 2025 Jun 2];19(2):125–133. Available from: https://www.nature.com/articles/ng0698_125 9620767 10.1038/477

[20] McCabe MJ , Alatzoglou KS , Dattani MT . Septo‐optic dysplasia and other midline defects: the role of transcription factors: HESX1 and beyond. Best Pract Res Clin Endocrinol Metab. 2011;25(1):115–124. 21396578 10.1016/j.beem.2010.06.008

[21] Salman MS , Ruth CA , Yogendran MS , Lix LM . Morbidities and comorbidities associated with optic nerve hypoplasia and septo‐optic‐pituitary dysplasia. Dev Med Child Neurol. 2025;67:941–952. 39804979 10.1111/dmcn.16235

[22] Guo H , Zhang Q , Dai R , Yu B , Hoekzema K , Tan J , et al. NCKAP1 disruptive variants Lead to a neurodevelopmental disorder with Core features of autism. Am J Hum Genet [Internet]. 2020 Nov [cited 2025 Jun 2];107(5):963–976. Available from: https://linkinghub.elsevier.com/retrieve/pii/S0002929720303608 33157009 10.1016/j.ajhg.2020.10.002 PMC7674997

[23] Tokita MJ , Braxton AA , Shao Y , Lewis AM , Vincent M , Küry S , et al. De novo truncating variants in SON cause intellectual disability, congenital malformations, and failure to thrive. Am J Hum Genet [Internet]. 2016 Sep [cited 2025 Jun 3];99(3):720–727. Available from: https://linkinghub.elsevier.com/retrieve/pii/S0002929716302774 27545676 10.1016/j.ajhg.2016.06.035 PMC5011061

[24] Slezak R , Smigiel R , Rydzanicz M , Pollak A , Kosinska J , Stawinski P , et al. Phenotypic expansion in Zhu‐Tokita‐Takenouchi‐Kim syndrome caused by de novo variants in the SON gene. Mol Genet Genomic Med. 2020;8(10):e1432. 32705777 10.1002/mgg3.1432 PMC7549597

[25] Dingemans AJM , Truijen KMG , Kim JH , Alaçam Z , Faivre L , Collins KM , et al. Establishing the phenotypic spectrum of ZTTK syndrome by analysis of 52 individuals with variants in SON. Eur J Hum Genet. 2022;30(3):271–281. 34521999 10.1038/s41431-021-00960-4 PMC8904542

[26] Okamoto N , Tsuchiya Y , Miya F , Tsunoda T , Yamashita K , Boroevich KA , et al. A novel genetic syndrome with STARD9 mutation and abnormal spindle morphology. Am J Med Genet A. 2017;173(10):2690–2696. 28777490 10.1002/ajmg.a.38391

[27] Senese S , Cheung K , Lo YC , Gholkar AA , Xia X , Wohlschlegel JA , et al. A unique insertion in STARD9's motor domain regulates its stability. Mol Biol Cell. 2015;26(3):440–452. 25501367 10.1091/mbc.E14-03-0829 PMC4310736

[28] Depaulis A , David O , Charpier S . The genetic absence epilepsy rat from Strasbourg as a model to decipher the neuronal and network mechanisms of generalized idiopathic epilepsies. J Neurosci Methods [Internet]. 2016 Feb [cited 2025 Jun 3];260:159–174. Available from: https://linkinghub.elsevier.com/retrieve/pii/S0165027015002137 26068173 10.1016/j.jneumeth.2015.05.022

[29] McCafferty C , David F , Venzi M , Lőrincz ML , Delicata F , Atherton Z , et al. Cortical drive and thalamic feed‐forward inhibition control thalamic output synchrony during absence seizures. Nat Neurosci [Internet]. 2018 May [cited 2025 Jun 3];21(5):744–756. Available from: https://www.nature.com/articles/s41593‐018‐0130‐4 29662216 10.1038/s41593-018-0130-4 PMC6278913

[30] International League Against Epilepsy Consortium on Complex Epilepsies . GWAS meta‐analysis of over 29,000 people with epilepsy identifies 26 risk loci and subtype‐specific genetic architecture. Nat Genet. 2023;55(9):1471–1482. 37653029 10.1038/s41588-023-01485-w PMC10484785

[31] Canela‐Xandri O , Rawlik K , Tenesa A . An atlas of genetic associations in UK biobank. Nat Genet [Internet]. 2018 Nov [cited 2025 Jun 3];50(11):1593–1599. Available from: https://www.nature.com/articles/s41588‐018‐0248‐z 30349118 10.1038/s41588-018-0248-z PMC6707814

[32] Devinsky O , Elder C , Sivathamboo S , Scheffer IE , Koepp MJ . Idiopathic generalized epilepsy: misunderstandings, challenges, and opportunities. Neurology [Internet]. 2024 Feb 13 [cited 2025 Jun 3];102(3):e208076. 10.1212/WNL.0000000000208076 38165295 PMC11097769

[33] Epi25 Collaborative . Ultra‐rare genetic variation in the epilepsies: a whole‐exome sequencing study of 17,606 individuals. Am J Hum Genet. 2019;105(2):267–282. 31327507 10.1016/j.ajhg.2019.05.020 PMC6698801

[34] Mullen SA , Marini C , Suls A , Mei D , Della Giustina E , Buti D , et al. Glucose transporter 1 deficiency as a treatable cause of myoclonic astatic epilepsy. Arch Neurol [Internet]. 2011 Sep 1 [cited 2024 Oct 30];68(9):1152. Available from: https://jamanetwork.com/journals/jamaneurology/fullarticle/1107863 21555602 10.1001/archneurol.2011.102

[35] Arsov T , Mullen SA , Rogers S , Phillips AM , Lawrence KM , Damiano JA , et al. Glucose transporter 1 deficiency in the idiopathic generalized epilepsies. Ann Neurol. 2012;72(5):807–815. 23280796 10.1002/ana.23702

[36] Carvill GL , Heavin SB , Yendle SC , McMahon JM , O'Roak BJ , Cook J , et al. Targeted resequencing in epileptic encephalopathies identifies de novo mutations in CHD2 and SYNGAP1. Nat Genet [Internet]. 2013 Jul [cited 2024 Oct 30];45(7):825–830. Available from: https://www.nature.com/articles/ng.2646 23708187 10.1038/ng.2646 PMC3704157

[37] Vlaskamp DRM , Shaw BJ , Burgess R , Mei D , Montomoli M , Xie H , et al. *SYNGAP1* encephalopathy: a distinctive generalized developmental and epileptic encephalopathy. Neurology [Internet]. 2019 Jan 8 [cited 2024 Oct 30];92(2):e96–e107. 10.1212/WNL.0000000000006729 30541864 PMC6340340

[38] Guerrini R , Scheffer I , Balestrini S . Epilepsy with myoclonic‐atonic seizures: an update on genetic causes, nosological limits, and treatment strategies. Lancet Neurol. 2025;24(4):348–360. 40120618 10.1016/S1474-4422(25)00032-8

[39] Lennox WG . The petit mal epilepsies: their treatment with TRIDIONE. JAMA. 1945;129(16):1069. 10.1001/jama.1945.02860500001001 21004764

[40] Loiseau P , Pestre M , Dartigues JF , Commenges D , Barberger‐Gateau C , Cohadon S . Long‐term prognosis in two forms of childhood epilepsy: typical absence seizures and epilepsy with rolandic (centrotemporal) EEG foci. Ann Neurol. 1983;13(6):642–648. 6410975 10.1002/ana.410130610

[41] Gurgu RS , Ciobanu AM , Danasel RI , Panea CA . Psychiatric comorbidities in adult patients with epilepsy (a systematic review). Exp Ther Med. 2021;22(2):909. 34249153 10.3892/etm.2021.10341 PMC8264824

[42] Chiron C , Marchand M , Tran A , Rey E , d'Athis P , Vincent J , et al. Stiripentol in severe myoclonic epilepsy in infancy: a randomised placebo‐controlled syndrome‐dedicated trial. Lancet [Internet]. 2000 Nov [cited 2021 Sep 4];356(9242):1638–1642. Available from: https://linkinghub.elsevier.com/retrieve/pii/S0140673600031573 11089822 10.1016/s0140-6736(00)03157-3

[43] Lagae L , Sullivan J , Knupp K , Laux L , Polster T , Nikanorova M , et al. Fenfluramine hydrochloride for the treatment of seizures in Dravet syndrome: a randomised, double‐blind, placebo‐controlled trial. Lancet. 2019;394(10216):2243–2254. 31862249 10.1016/S0140-6736(19)32500-0

[44] Nabbout R , Mistry A , Zuberi S , Villeneuve N , Gil‐Nagel A , Sanchez‐Carpintero R , et al. Fenfluramine for treatment‐resistant seizures in patients with Dravet syndrome receiving Stiripentol‐inclusive regimens: a randomized clinical trial. JAMA Neurol [Internet]. 2020 Mar 1 [cited 2021 Jan 17];77(3):300. Available from: https://jamanetwork.com/journals/jamaneurology/fullarticle/2756124 31790543 10.1001/jamaneurol.2019.4113 PMC6902175

[45] Balestrini S , Chiarello D , Gogou M , Silvennoinen K , Puvirajasinghe C , Jones WD , et al. Real‐life survey of pitfalls and successes of precision medicine in genetic epilepsies. J Neurol Neurosurg Psychiatry [Internet]. 2021 Apr 26 [cited 2021 May 23];92(10):1044–1052. 10.1136/jnnp-2020-325932 33903184 PMC8458055

