# Supplementary material for: Clinical and genetic landscape of epilepsies with absence seizures and single‐gene etiology
Source: Epilepsia. 2025 Oct 25;67(1):272–90. doi: 10.1111/epi.18655 (PMC12893263; doi:10.1111/epi.18655)
Supplement: Supplementary file 1 — Figure S1. [file EPI-67-272-s003.docx]

**Supplementary Figure 1. Representative ictal EEG traces from two patients with absence seizure onset before 1 year of age.**

(A) Two clinical events recorded in a patient aged 7 months with an *SLC2A1* pathogenic variant. The top panel shows irregular spike–wave and slow-wave activity with frontal predominance, lasting 8 seconds, recorded during drowsiness. The bottom panel shows a more generalized 3 Hz spike–wave discharge lasting 5 seconds during wakefulness. During both events, the patient exhibited upward eye deviation and behavioural arrest. Recording parameters: high-pass filter: 1,600 Hz; low-pass filter 30 Hz; gain 250 μV/cm (top panel), 200 μV/cm (bottom panel).


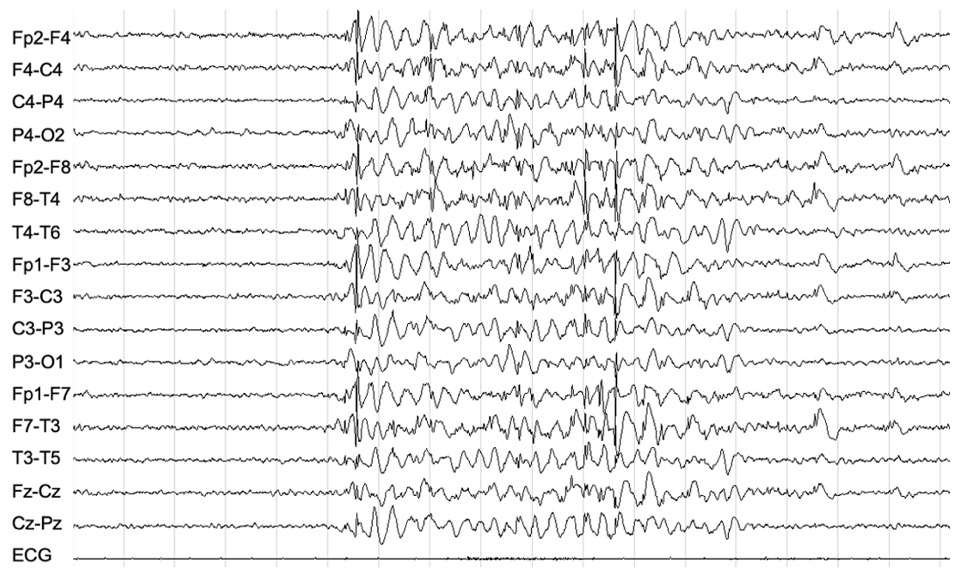


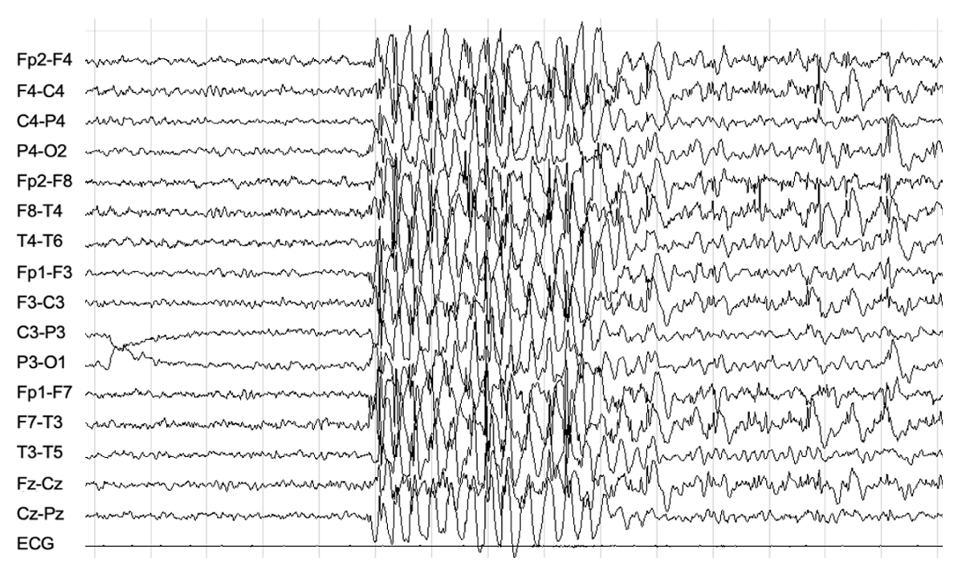


(B) Brief clinical event recorded in a patient aged 10 months with a likely pathogenic *SCN1A* variant. 25 Hz photic stimulation induces an irregular high amplitude spike-wave discharge, lasting 3 seconds, which is accompanied, clinically, by upward eye deviation and behavioural arrest. Recording parameters: high-pass filter: 1,600 Hz; low-pass filter 30 Hz; gain 250 μV/cm; notch filter 50 Hz.


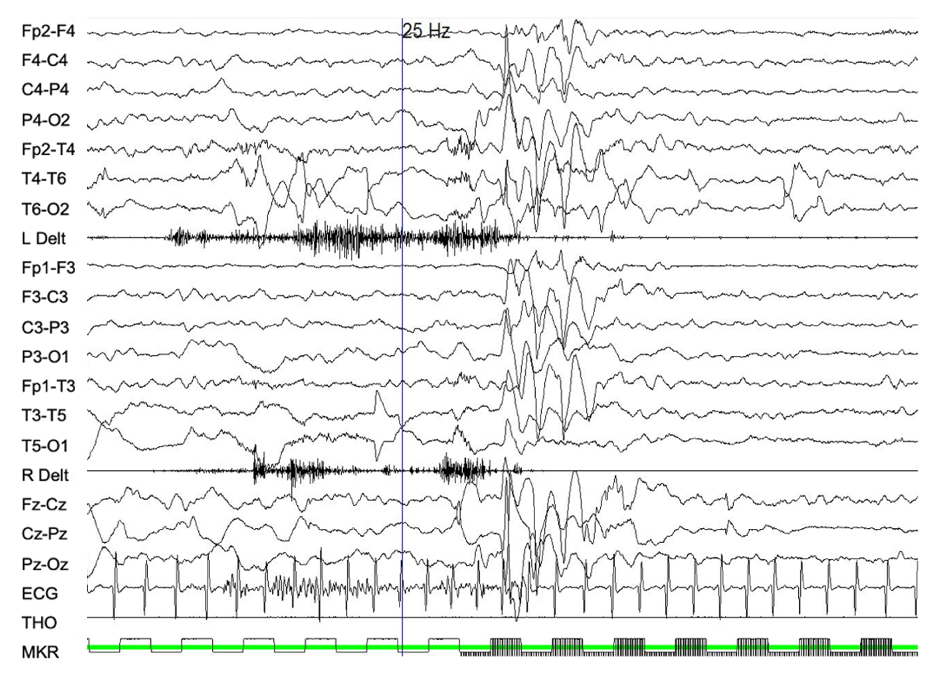


*ECG = electrocardiogram; L Delt* = left deltoid; *MKR*= time marker (1 second); *R Delt* = right deltoid; *THO = thoracic respiratory effort (not active here)*
