# Supplementary material for: Clinical and genetic landscape of epilepsies with absence seizures and single‐gene etiology
Source: Epilepsia. 2025 Oct 25;67(1):272–90. doi: 10.1111/epi.18655 (PMC12893263; doi:10.1111/epi.18655)
Supplement: Supplementary file 2 — Figure S2. [file EPI-67-272-s002.docx]

**Supplementary Figure 2. Representative ictal EEG traces from three patients with absence seizures precipitated by eye closure and intermittent photic stimulation (IPS).**

1. Episode of behavioural arrest and eyelid myoclonia precipitated by eye closure in a patient aged 6 years with a pathogenic *SETD1B* variant. Two seconds after eye closure, the EEG shows bilateral diffuse high amplitude spike-wave and slow-wave activity with frontal predominance and irregular frequency, lasting 6 seconds. IPS (as recognizable in the MKR channel) is triggered at the end of the discharge and has no effect on it. Recording parameters: high-pass filter: 1,800 Hz; low-pass filter 30 Hz; gain 250 μV/cm; notch filter 50 Hz.


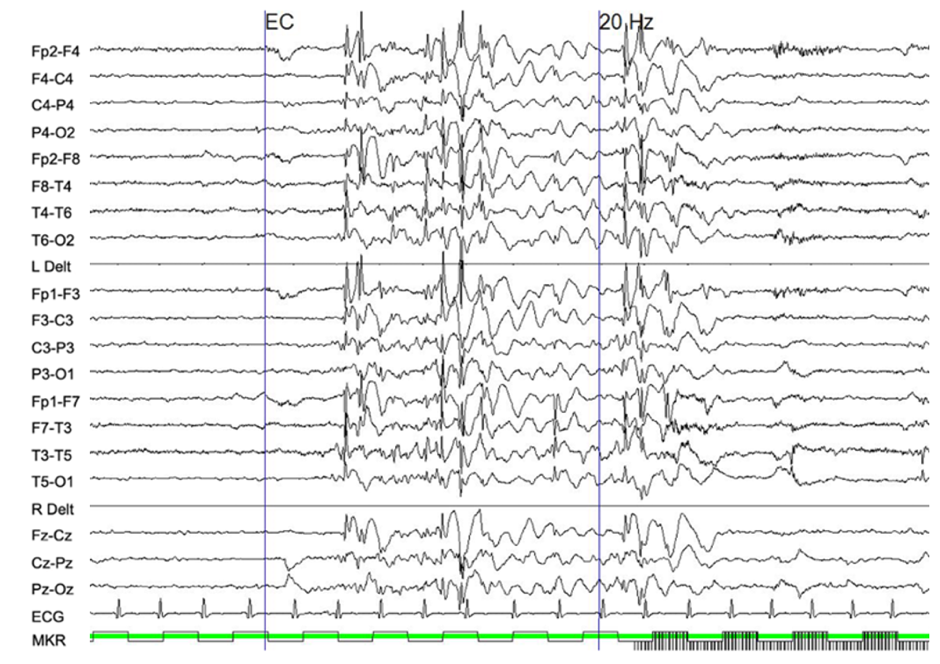


1. Absence seizure recorded in a patient aged 22 years with a pathogenic *NARS1* variant. 35Hz photic stimulation induces an irregular, diffuse spike–wave discharge, with fronto-central predominance, lasting 5 seconds. The patient exhibits upward eye deviation and behavioural arrest. Recording parameters: high-pass filter: 1,600 Hz; low-pass filter 30 Hz; gain 250 μV/cm; notch filter 50 Hz.

**
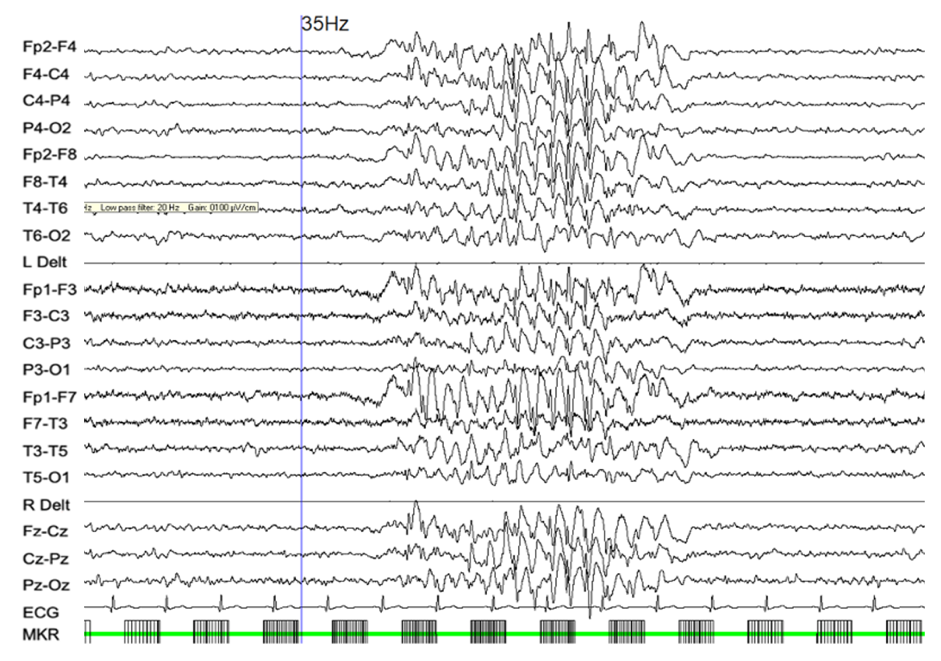
**

1. Absence seizure recorded in a patient aged 7 years with a likely pathogenic *SEMA6B* variant. When incremental frequency photic stimulation reaches 20Hz, an irregular spike–wave discharge, with fronto-central predominance, lasting 6 seconds, is observed. During the discharge, the patient loses his facial expression and exhibits behavioural arrest. Recording parameters: high-pass filter: 1,600 Hz; low-pass filter 30 Hz; gain 200 μV/cm; notch filter 50 Hz.

**
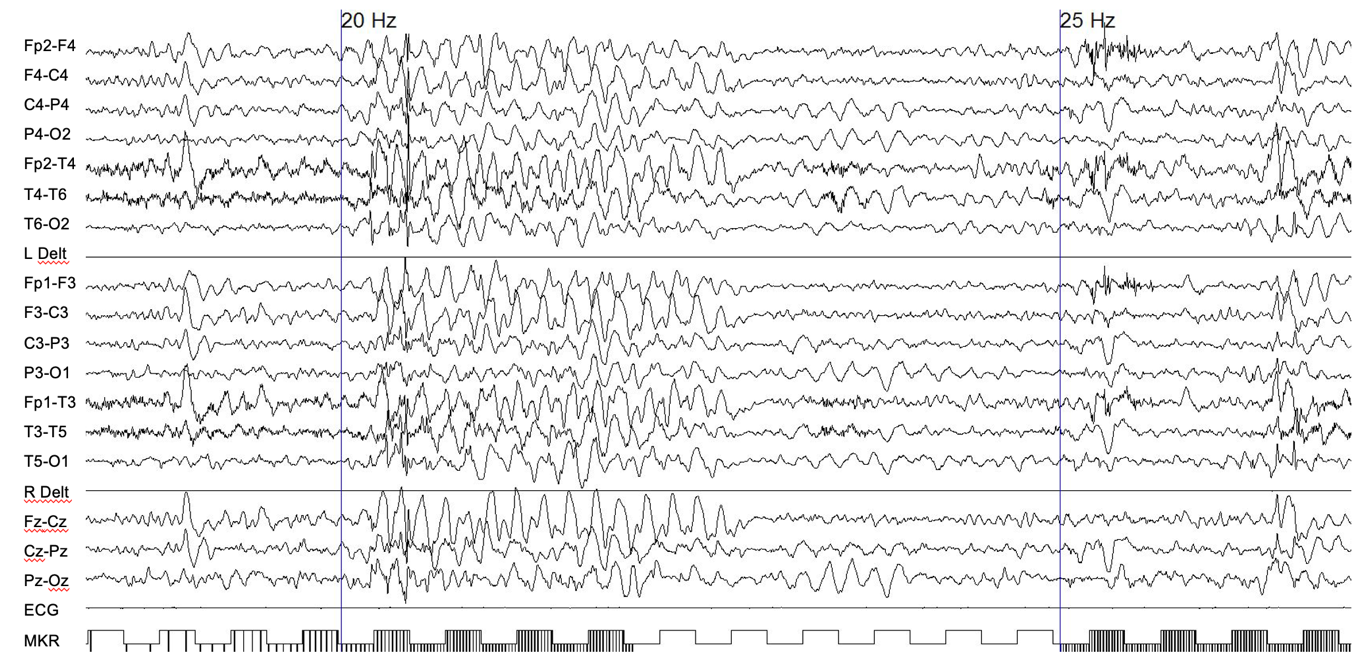
**

*EC= eyes closure; ECG= electrocardiogram; L Delt* = left deltoid; *MKR*= time marker and IPS signal (1 second); *R Delt* = right deltoid.
