# Supplementary material for: Clinical and genetic landscape of epilepsies with absence seizures and single‐gene etiology
Source: Epilepsia. 2025 Oct 25;67(1):272–90. doi: 10.1111/epi.18655 (PMC12893263; doi:10.1111/epi.18655)
Supplement: Supplementary file 3 — Figure S3. [file EPI-67-272-s001.docx]

**Supplementary Figure 3. Representative ictal EEG traces from three patients with non-convulsive status epilepticus.**

1. Non-convulsive status epilepticus in 9-year-old patient with a pathogenic *SCN1A* variant. There are continuous irregular spikes and spike-waves over both frontal regions. Clinically there is prolonged ideomotor slowing (lasting 2 hours and 40 minutes), with interspersed brief, episodes of motionlessness, unresponsiveness, and eyelid myoclonia. Recording parameters: high-pass filter: 1,600 Hz; low-pass filter 30 Hz; gain 250 μV/cm; notch filter 50 Hz.


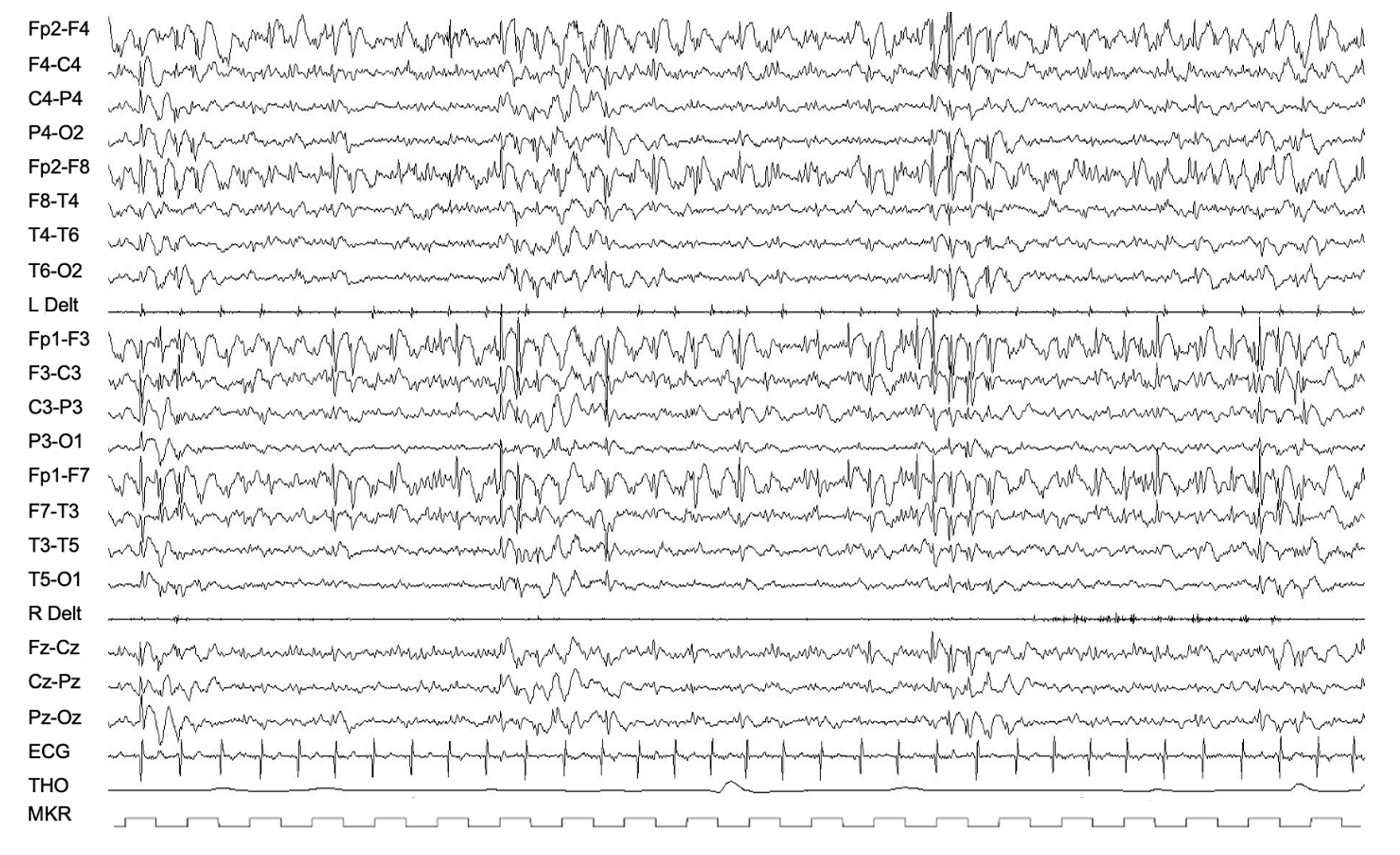


1. Non-convulsive status epilepticus in a patient aged 3 years with a likely pathogenic *KMT2E* variant. There is continuous irregular spike and spike-wave activity over both frontal regions. Prolonged absence status (30-40 minutes) during which the patient manifests ideomotor slowing and fluctuating unresponsiveness. Recording parameters: high-pass filter: 1,600 Hz; low-pass filter 30 Hz; gain 250 μV/cm; notch filter 50 Hz.


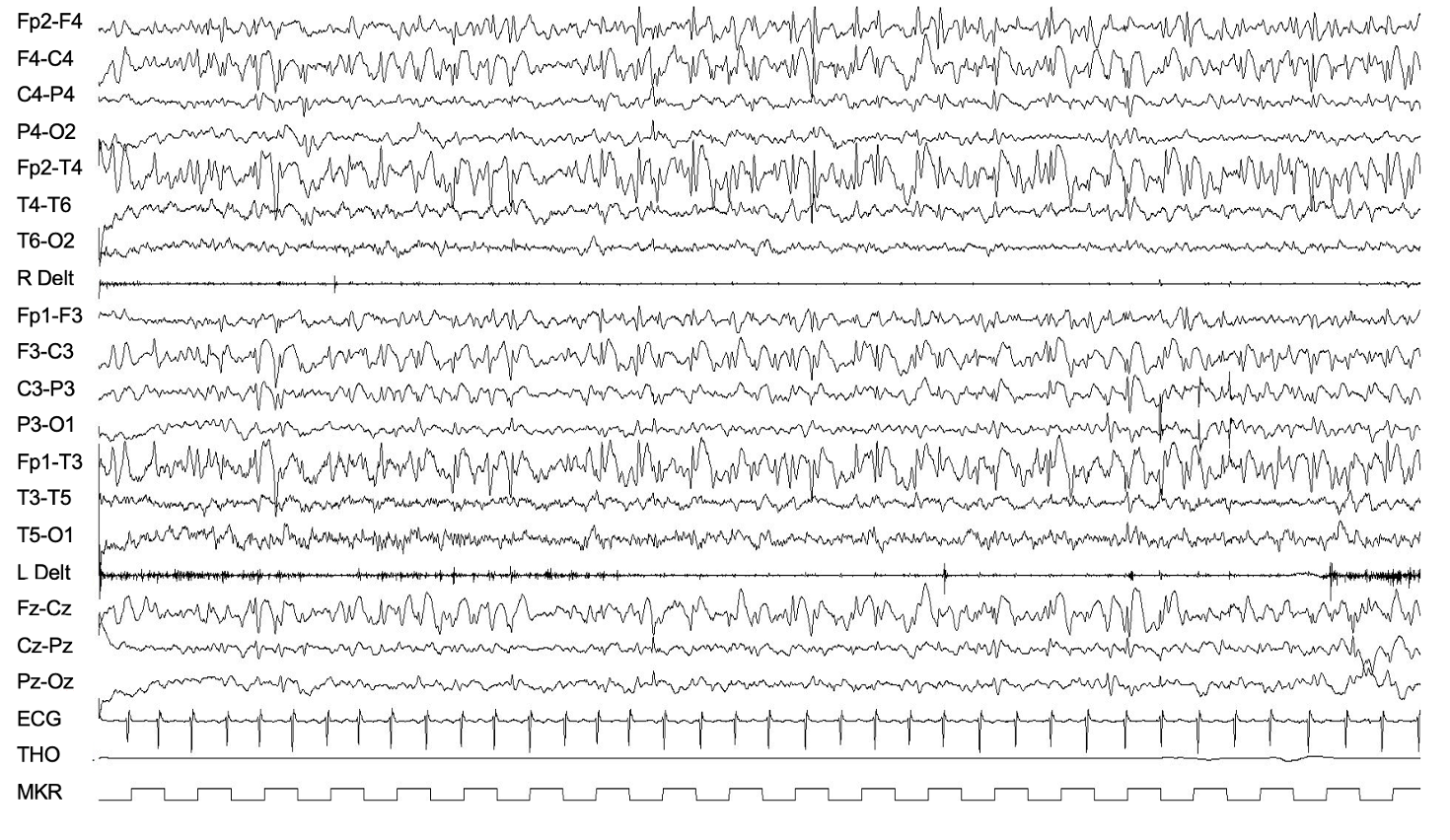


1. Non-convulsive status epilepticus in a patient aged 13 years with a likely pathogenic *SLC2A1* variant. There is subcontinuous irregular generalized spike and spike-wave activity, during which the child is confused and intermittently unresponsive. Recording parameters: high-pass filter: 1,600 Hz; low-pass filter 30 Hz; gain 200 μV/cm; notch filter 50 Hz.

**
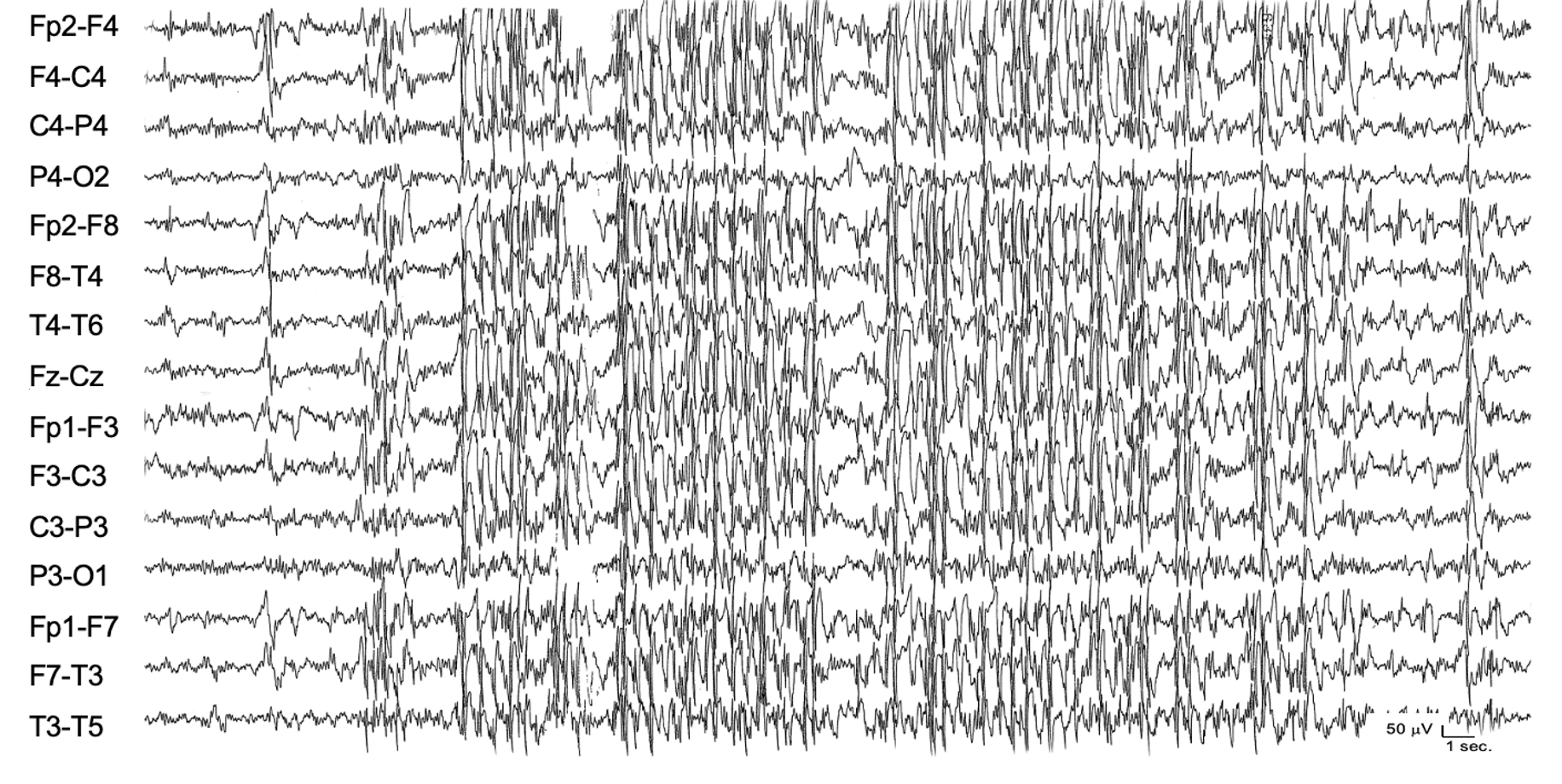
**

*ECG= electrocardiogram; L Delt* = left deltoid muscle; *MKR*= time marker (1 second); *R Delt* = right deltoid; *THO = thoracic respiratory effort (not active here).*
